# Supplementary material for: Pre-Treatment Biomarkers of Anti-Tumour Necrosis Factor Therapy Response in Crohn’s Disease—A Systematic Review and Gene Ontology Analysis
Source: Cells. 2019 May 28;8(6):515. doi: 10.3390/cells8060515 (PMC6628089; doi:10.3390/cells8060515)
Supplement: Supplementary file 1 [file cells-08-00515-s001.zip › Supplementary material- finalized-/Supplementary Figure S5.pdf]

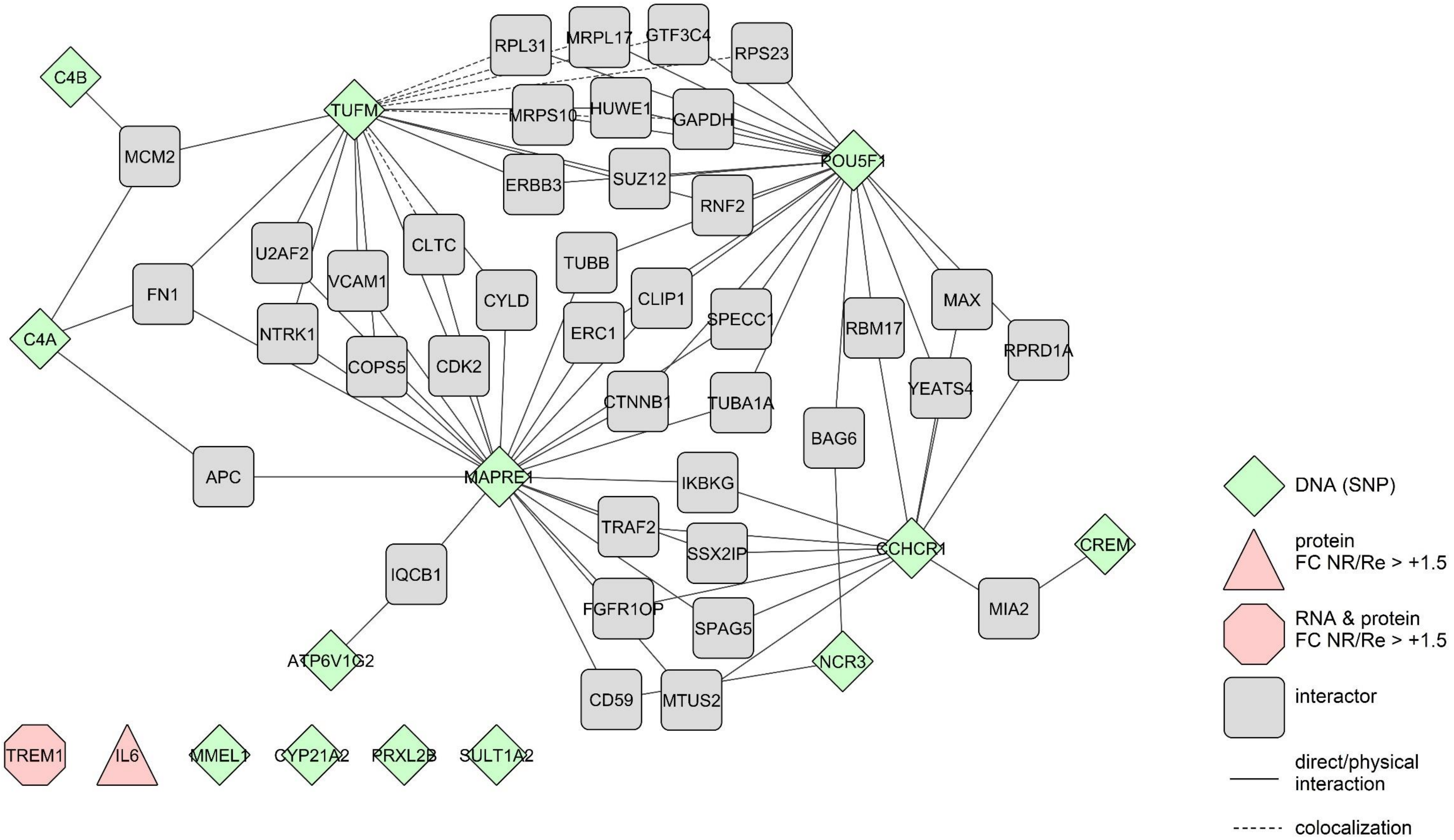

Supplementary Figure S5. Extended interactome of the integrated blood markers linked to the long-term anti-TNF therapy response in CD patients. Different forms represent markers measured at different levels of expression. The colours represent differences in expression level (FC- fold change) between therapy nonresponders (NR) and responders (Re). Lines represent direct protein-protein interactions.
