# Supplementary material for: Pre-Treatment Biomarkers of Anti-Tumour Necrosis Factor Therapy Response in Crohn’s Disease—A Systematic Review and Gene Ontology Analysis
Source: Cells. 2019 May 28;8(6):515. doi: 10.3390/cells8060515 (PMC6628089; doi:10.3390/cells8060515)
Supplement: Supplementary file 1 [file cells-08-00515-s001.zip › Supplementary material- finalized-/Supplementary methods.pdf]

cells

MDPI

Supplementary Methods

Information sources and search

We conducted the final search of the electronically available sources on the PubMed by crossing the following partial searches:

Search #1: Crohn's disease OR inflammatory bowel disease OR IBD

Search #2: anti-TNF OR anti-tumor necrosis factor OR anti-tumour necrosis factor OR TNF OR tumor necrosis factor OR tumour necrosis factor OR infliximab OR adalimumab OR golimumab OR entanercept OR certolizumab

Search #3: response OR responsiveness OR nonresponse OR non-response OR nonresponsiveness OR non-responsiveness OR outcome OR therapy OR treatment OR prediction OR predictive OR predicting

Final Search: Search #1 AND Search #2 AND Search #3 AND English [Language]

We searched for all the above terms as “Title/Abstract” and “Text” words. Appropriate MeSH terms were also included.

The full query translation was: ((((((((((((((((((((((((((((tumor necrosis factor[MeSH Terms]) OR tumor necrosis factor[Title/Abstract]) OR tumor necrosis factor[Text Word]) OR tumour necrosis factor[MeSH Terms]) OR tumour necrosis factor[Title/Abstract]) OR tumour necrosis factor[Text Word]) OR tnf alpha[MeSH Terms]) OR TNF[Text Word]) OR TNF[Title/Abstract]) OR anti-TNF[Text Word]) OR anti-TNF[Title/Abstract]) OR anti-tumor necrosis factor[Text Word]) OR anti-tumor necrosis factor[Title/Abstract]) OR anti-tumour necrosis factor[Text Word]) OR anti-tumour necrosis factor[Title/Abstract]) OR infliximab[Text Word]) OR infliximab[Title/Abstract]) OR adalimumab[Text Word]) OR adalimumab[Title/Abstract]) OR golimumab[Title/Abstract]) OR golimumab[Text Word]) OR entanercept[Title/Abstract]) OR entanercept[Text Word]) OR certolizumab[Title/Abstract]) OR certolizumab[Text Word])) AND ((((((((((((((((((((((((response[Text Word]) OR response[Title/Abstract]) OR responsiveness[Text Word]) OR responsiveness[Title/Abstract]) OR non-response[Text Word]) OR non-response[Title/Abstract]) OR nonresponse[Text Word]) OR nonresponse[Title/Abstract]) OR nonresponsiveness[Text Word]) OR nonresponsiveness[Title/Abstract]) OR non-responsiveness[Text Word]) OR non-responsiveness[Title/Abstract]) OR outcome[Text Word]) OR outcome[Title/Abstract]) OR therapy[Text Word]) OR therapy[Title/Abstract]) OR treatment[Text Word]) OR treatment[Title/Abstract]) OR prediction[Text Word]) OR prediction[Title/Abstract]) OR predictive[Text Word]) OR predictive[Title/Abstract]) OR predicting[Text Word]) OR predicting[Title/Abstract])) AND ((((((((crohn disease[MeSH Terms]) OR crohn's disease[Text Word]) OR crohn's disease[Title/Abstract]) OR inflammatory bowel disease[MeSH Terms]) OR inflammatory bowel disease[Text Word]) OR inflammatory bowel disease[Title/Abstract]) OR IBD[Text Word]) OR IBD[Title/Abstract])) AND "english"[Language]

Study selection

We performed study selection in several rounds. First, we automatically excluded publications not reporting novel measurements in patients, i.e. comments, editorials, meta-analysis, reviews and systematic reviews. Also excluded were studies with a sample size <5 (i.e. case reports) as well as retracted publications and retractions of publications.

For further selection, we screened the studies' titles and abstracts. We excluded obviously irrelevant studies, studies focusing solely on Ulcerose colitis and paediatric IBD and studies reporting only experimental (animal, *in vitro*) data.

We retrieved the remaining studies in full text and screened them further. We excluded publications reporting on pharmacokinetics (therapeutic drug monitoring), anti-therapeutic antibodies, treatment regimes, microbiota, patients' lifestyle and other response predictors other than genetic and expression.

In the final round of selection, we excluded studies reporting expression biomarkers measured after the therapy start, those not comparing responders to anti-TNF therapy and nonresponding patients (i.e. comparing responders/non-responders to untreated patients or healthy controls) and studies where data on CD patients could not be discriminated from IBD data.

Gene ontology (GO) analysis

We used CytoScape's ClueGO plug-in for GO analysis. We searched for EBI-UniProt-GOA GO ontologies and pathways (Molecular Function, Biological Process, Cellular Component and Immune System Process). We allowed all experimental evidence. Network specificity was set at medium. We made separated GO term selection of enriched and depleted terms with right-sided and left-sided hypergeometric test, respectively. We used Bonferroni step-down to correct the p-values for multiple testing. Level of significance was set at corrected $p<0.05$.
