# Supplementary material for: Pre-Treatment Biomarkers of Anti-Tumour Necrosis Factor Therapy Response in Crohn’s Disease—A Systematic Review and Gene Ontology Analysis
Source: Cells. 2019 May 28;8(6):515. doi: 10.3390/cells8060515 (PMC6628089; doi:10.3390/cells8060515)
Supplement: Supplementary file 1 [file cells-08-00515-s001.zip › Supplementary material- finalized-/Supplementary tables- final corrected BG.pdf]

cells MDPI

Supplementary Table S1. Publications included in the systematic review.

reference	patients included	therapy used	response definition	response	tissue	data type	methods	data extracted	PMID
[55]	75	IFX	CDAI, IBDQ	Short-term	N.D.	DNA	allele-specific hybridization	no	PMID:11313304
[57]	90	IFX	CDAI	Short-term	blood	DNA	allele-specific PCR	yes	PMID:12049175
[63]	254	IFX	CDAI, cl.ass.	Short-term	N.D.	DNA	allele-specific PCR	no	PMID:12105838
[34]	153	IFX	CDAI	Short-term	serum	protein	ELISA	yes	PMID:12190096
	226				blood	DNA	allele-specific PCR		
[45]	36	IFX	cl.ass.	Short-term	serum	protein	ELISA	yes	PMID:12358255
[64]	534	IFX	CDAI	Short-term	blood	DNA	allele-specific PCR	no	PMID:12360101
[23]	200	IFX	CDAI	Short-term	N.D.	DNA	allele-specific PCR	yes	PMID:14987319
[65]	166	IFX	CDAI	Short-term	blood	DNA	PCR-RFLP	yes	PMID:15274667
[28]	40	IFX	CDAI	Short-term	N.D.	DNA	allele-specific PCR	yes	PMID:15754402
[21]	259	IFX	CDAI	Short-term	PBMCs	DNA	PCR-RFLP	yes	PMID:16181301
[66]	478	IFX	cl.ass.	Short-term	PBMCs	DNA	PCR-DHPLC	no	PMID:16305727
[67]	206	IFX	CDAI	Short-term	blood	DNA	allele-specific PCR, RFLP	no	PMID:16344720
[22]	214	IFX	CDAI	Short-term	N.D.	DNA	allele-specific PCR, sequencing	no	PMID:16609369
[68]	222	IFX	CDAI	Short-term	N.D.	DNA	allele-specific PCR, sequencing	yes	PMID:17001292
[69]	344	IFX	CDAI	Short-term	N.D.	DNA	allele-specific PCR	no	PMID:17108815
[70]	265	IFX	CDAI	Short-term	blood	DNA	allele-specific PCR	no	PMID:17505995
[71]	80	IFX	HBI	Short-term	PBMCs	DNA	allele-specific hybridization	yes	PMID:18248655
[72]	20	IFX	CDAI	Short-term	serum	protein	SELDI-TOF, ELISA	no	PMID:18489908
[73]	199	IFX	HBI	Short-term	blood	DNA	allele-specific PCR, PCR-RFLP	no	PMID:19693669
[12]	37	IFX	cl.ass.	Short-term	colon mucosa	RNA	microarray	yes	PMID:19956723
[42]	40	IFX	CDAI	Short-term	colon mucosa	protein	IHC	no	PMID:20188102
					serum		ELISA	yes	
[44]	20	IFX	CDAI	Short-term	serum	protein	ELISA	yes	PMID:20848485
[30]	37	IFX	cl.ass.	Short-term	colon mucosa	RNA	microarray, qRT-PCR	yes	PMID:20848504
[74]	268	IFX	cl.ass.	Long-term	serum	protein	ELISA	no	PMID:21334460
[75]	185	IFX / ADM	CDAI	Short-term	PBMCs	DNA	sequencing	no	PMID:22147245
[35]	385	IFX	CDAI	Short-term	serum	protein	Immunoturbidi-metric assay	yes	PMID:22251435

[43]	35	IFX	CDAI	Short-term	serum	protein	ELISA	yes	PMID:22398057
[76]	26	ADM	CDAI, HBI	Long-term<br>Short-term	serum	protein	ELISA	no	PMID:22784949
[26]	104	IFX	cl.ass.	Short-term	blood	DNA	multiplex bead-based assay	yes	PMID:22860894
[27]	29	IFX	CDAI	Short-term	blood	DNA	allele-specific PCR, sequencing	yes	PMID:22960943
[77]	65	IFX	CDAI	Short-term	colon mucosa<br>serum	RNA<br>protein	qRT-PCR<br>ELISA	no	PMID:23039249
[78]	48	IFX	CDAI	Long-term	PBMCs	DNA	SNaPshot, sequencing	no	PMID:23049214
[79]	102	IFX	CDAI	Short-term	PBMCs	DNA	allele-specific PCR	no	PMID:23358932
[80]	179	IFX / ADM	CDAI	Long-term	PBMCs<br>serum	DNA<br>protein	allele-specific PCR<br>ELISA	no	PMID:23376290
[31]	20	IFX	CDAI	Short-term	PBMCs	RNA	microarray	yes	PMID:23809696
[36]	148	IFX	HBI	Short-term	serum	protein	ELISA	yes	PMID:23932786
[25]	297	IFX	HBI	Short-term	N.D.	DNA	allele-specific PCR	yes	PMID:24121042
[54]	43	IFX / ADM	HBI	Short-term	serum	protein	TRIFMA, ELISA	no	PMID:24291022
[40]	25	ADM	CDAI	Short-term	colon mucosa	protein	IFM	yes	PMID:24562382
[24]	126	IFX	HBI	Short-term	serum<br>blood	protein<br>DNA	N.D.<br>allele-specific PCR	no<br>yes	PMID:24707144
[56]	106	IFX	HBI, cl.ass.	Short-term	blood	DNA	allele-specific PCR	no	PMID:24714240
[49]	782	anti-TNF	simple 3-step scale	Short-term	blood	DNA	allele-specific PCR	yes	PMID:24776844
[81]	32	anti-TNF	HBI	Short-term	serum	protein	ELISA	no	PMID:25625487
[82]	19	IFX	CDAI	Short-term	serum	protein	CE-LIF	no	PMID:25639738
[83]	35	anti-TNF	N.D.	Short-term	colon mucosa	protein	IHC	no	PMID:25699367
[7]	97	ADM	IBDQ	Long-term<br>Short-term	PBMCs	DNA	PCR-RFLP	yes	PMID:25712183
[38]	33	IFX	HBI	Short-term	stool	protein	ELISA	yes	PMID:25811118
[84]	87	IFX	HBI	Short-term	blood	DNA	PCR-RFLP	no	PMID:25918954
[46]	39	IFX	CDAI	Long-term	serum	protein	latex agglutination immunoassay, ELISA	yes	PMID:26218144
[85]	350	IFX	HBI	Short-term	N.D.	DNA	allele-specific PCR	yes	PMID:26339133
[48]	163<br>201	IFX	cl.ass.	Short-term	N.D.<br>serum	DNA<br>protein	immunochip<br>N.D.	no	PMID:26351386
[86]	103	IFX	CDAI	Long-term	blood	DNA	PCR-RFLP	yes	PMID:26558270
[39]	30	ADM	HBI	Short-term	stool	protein	ELISA	yes	PMID:26595391
[8]	79	ADM	IBDQ	Long-term<br>Short-term	PBMCs	DNA	allele-specific PCR	yes	PMID:27096233

[9]	68	ADM	IBDQ	Long-term	serum	protein	N.D.	no	PMID:27115882
				Short-term	PBMCs	DNA	allele-specific PCR	yes	
					serum	protein	N.D.	no	
[10]	359	IFX / ADM / CZP	cl.ass.	Long-term<br>Short-term	PBMCs	DNA	immunochip	yes	PMID:27596696
[87]	482	anti-TNF	simple 3-step scale	Short-term	blood	DNA	allele-specific PCR	yes	PMID:28139755
[11]	314	IFX / ADM / CZP	cl.ass.	Short-term	PBMCs	DNA	immunochip	yes	PMID:28590340
[41]	201	IFX	cl.ass.	Short-term	serum	protein	ELISA	yes	PMID:28622097
[47]	121	IFX / ADM / CZP	cl.ass.	Long-term	blood	DNA	allele-specific PCR	yes	PMID:28785150
[88]	107	IFX / ADM	HBI, cl.ass.	Short-term	blood	DNA	PCR-RFLP	no	PMID:29152405
[32]	22	IFX	HBI	Short-term	PBMCs	RNA	microarray	yes	PMID:29618496
[13]	19	IFX / ADM	CDAI, SES-CD	Short-term	colon mucosa	RNA	qRT-PCR	no	PMID:29848778
						protein	IFM		
[33]	85	IFX / ADM	cl.ass.	Long-term	PBMCs	RNA	qRT-PCR	yes	PMID:30007919
					serum	protein	ELISA		
[37]	35	IFX	HBI	Short-term	stool	protein	ELISA	yes	PMID:30149385
					serum		N.D.		
[29]	121	IFX	CDAI	Long-term	blood	DNA	allele-specific PCR	yes	PMID:30286108

Therapy used: ADM (adalimumab), anti-TNF (unindetified anti-TNF biological), CZP (certolizumab pegol), IFX (infliximab). **Response definition:** CDAI (Crohns's Disease Activity Index), IBDQ (Inflammatory Bowel Disease Questionnaire), HBI (Harwey-Bradshaw Index), SES-CD (Simple Endoscopic Score for Crohn's Disease), cl.ass. (clinical assessment). **Methods:** PCR-RFLP (PCR-Restriction Fragment Lenght Polymorhism), PCR-DHPLC (Denaturing High-Performance Liquid Chromatography), qRT-PCR (quantitatitve real-time PCR), SELDI-TOF (Surface-Enhancved Laser Desorption/Ionozation- Time-of-Flight mass spectometry), ELISA (Enzyme-Linked Immunosorbent Assay), IHC (Immunohistochemistry), IFM (Immunofluorescent Microscopy), TRIFMA (Time-Resolved Immunofluorometric Assay), CE-LIF (Capillary Electrophoresis-Laser-Induced Fluorescence). **N.D.** (not defined).

Supplementary Table S2. SNPs linked to short-term anti-TNF response in CD patients.

SNP	p-value	adjusted p-value	allele / genotype association		ref.
rs704191	1.70×10^{-5}	*$1.52 \times 10^{+0}$*	A	NR	[11]
rs8083571	2.40×10^{-5}	*$4.72 \times 10^{+0}$*	A	NR	[10]
rs1130864	4.09×10^{-5}	*4.09×10^{-4}*	C	Re	[8]
rs7956809	4.30×10^{-5}	*$8.45 \times 10^{+0}$*	G	NR	[10]
rs17200795	4.60×10^{-5}	*$9.04 \times 10^{+0}$*	G	NR	[10]
rs10456777	6.10×10^{-5}	*$5.46 \times 10^{+0}$*	C	NR	[11]
rs2045307	7.40×10^{-5}	*$1.45 \times 10^{+1}$*	C	NR	[10]
rs10210302	8.10×10^{-4}	2.67×10^{-2}	CC	Re	[7]
rs9373839	1.13×10^{-3}	*1.13×10^{-2}*	C	Re	[8]
rs4845604	1.90×10^{-3}	*$3.73 \times 10^{+2}$*	A	NR	[10]
rs1813443	2.00×10^{-3}	6.00×10^{-3}	CC	NR	[24]
rs976881	2.00×10^{-3}	*2.40×10^{-2}*	A	NR	[26]
**rs396991**	3.00×10^{-3}	3.00×10^{-3}	GG	Re	[23]
	2.10×10^{-2}	6.93×10^{-1}	GG	Re	[7]
rs10512734	4.60×10^{-3}	1.52×10^{-1}	A	Re	[7]
rs1061624	5.00×10^{-3}	*2.00×10^{-2}*	A	NR	[25]
rs8049439	5.60×10^{-3}	1.85×10^{-1}	C	Re	[7]
rs7927894	5.80×10^{-3}	1.91×10^{-1}	CC	Re	[7]
rs1295686	6.10×10^{-3}	2.01×10^{-1}	AA	Re	[7]
rs12777960	6.70×10^{-3}	2.21×10^{-1}	A	Re	[7]
**rs1061622**	7.00×10^{-3}	*8.40×10^{-2}*	G	Re	[26]
	3.60×10^{-2}	*3.96×10^{-1}*	G	NR	[57]
rs1568885	7.00×10^{-3}	2.10×10^{-2}	TT	NR	[24]
**rs4645983**	7.20×10^{-3}	2.38×10^{-1}	C	Re	[7]
	N.D.	3.00×10^{-2}	TT	Re	[21]
rs2071303	8.20×10^{-3}	*1.64×10^{-2}*	G	Re	[9]
rs3804099	1.00×10^{-2}	*3.90×10^{-1}*	TC/CC	Re	[49]
rs5744174	1.00×10^{-2}	*$1.83 \times 10^{+0}$*	TC	Re	[87]
rs945439	1.00×10^{-2}	*1.10×10^{-1}*	G	NR	[57]
rs9297145	1.10×10^{-2}	*$9.84 \times 10^{+2}$*	C	NR	[11]
rs3748816	1.20×10^{-2}	3.96×10^{-1}	C	Re	[7]
rs9847710	1.30×10^{-2}	*$2.55 \times 10^{+3}$*	C	NR	[10]
rs1182188	1.40×10^{-2}	*$2.75 \times 10^{+3}$*	C	NR	[10]
rs3024505	1.40×10^{-2}	4.62×10^{-1}	C	Re	[7]
rs3774934	1.40×10^{-2}	*1.40×10^{-1}*	A	Re	[8]
rs7517847	1.60×10^{-2}	5.28×10^{-1}	GG	Re	[7]
rs11677200	1.70×10^{-2}	*2.04×10^{-1}*	CC	Re	[85]
rs8126756	2.00×10^{-2}	*$3.66 \times 10^{+0}$*	CC	Re	[87]
rs10509115	2.20×10^{-2}	7.26×10^{-1}	A	Re	[7]
rs11575934	2.20×10^{-2}	7.26×10^{-1}	AA	Re	[7]
rs3766606	2.20×10^{-2}	*$4.32 \times 10^{+3}$*	T	NR	[10]
rs1050152	2.40×10^{-2}	7.92×10^{-1}	C	Re	[7]
rs2522057	2.40×10^{-2}	*4.80×10^{-2}*	C	NR	[28]
rs35260072	2.40×10^{-2}	*4.80×10^{-2}*	G	NR	[28]
rs2476601	2.50×10^{-2}	8.25×10^{-1}	A	Re	[7]
rs3197999	2.50×10^{-2}	*$4.91 \times 10^{+3}$*	A	NR	[10]
rs2631372	2.60×10^{-2}	8.58×10^{-1}	CC+CG	Re	[7]
rs4263839	2.70×10^{-2}	8.91×10^{-1}	AA	Re	[7]
rs3087243	2.80×10^{-2}	9.24×10^{-1}	AA	Re	[7]
rs2430561	3.00×10^{-2}	*$1.17 \times 10^{+0}$*	TA/AA	Re	[49]
rs5030728	3.00×10^{-2}	*$1.17 \times 10^{+0}$*	GA/AA	Re	[49]
**rs767455**	3.40×10^{-2}	*6.80×10^{-2}*	G	NR	[65]
	4.00×10^{-2}	*2.00×10^{-1}*	G	NR	[71]
rs1800682	3.50×10^{-2}	$1.16 \times 10^{+0}$	C	Re	[7]
rs2070874	3.60×10^{-2}	$1.19 \times 10^{+0}$	C	Re	[7]
rs2872507	3.60×10^{-2}	$1.19 \times 10^{+0}$	A	Re	[7]
rs11465996	4.00×10^{-2}	*$1.56 \times 10^{+0}$*	CG/GG	Re	[49]
rs3397	4.90×10^{-2}	*1.96×10^{-1}*	CC	Re	[25]
rs3814057	4.00×10^{-2}	$1.32 \times 10^{+0}$	AA+AC	Re	[7]
rs4149570	4.00×10^{-2}	*$1.56 \times 10^{+0}$*	TT	Re	[49]
rs2503322	4.10×10^{-2}	*$8.06 \times 10^{+3}$*	A	NR	[10]
rs13294	4.50×10^{-2}	$1.49 \times 10^{+0}$	AA+A	Re	[7]
rs1728785	4.50×10^{-2}	*$8.84 \times 10^{+3}$*	A	NR	[10]
rs6708413	4.50×10^{-2}	*$8.84 \times 10^{+3}$*	G	NR	[10]
rs6887695	4.50×10^{-2}	$1.49 \times 10^{+0}$	C	Re	[7]
rs10761659	4.70×10^{-2}	*$9.24 \times 10^{+3}$*	A	NR	[10]
rs4246905	4.70×10^{-2}	*$9.24 \times 10^{+3}$*	T	NR	[10]
rs2066845	4.90×10^{-2}	$1.62 \times 10^{+0}$	C	Re	[7]
rs921720	4.90×10^{-2}	*$9.63 \times 10^{+3}$*	A	NR	[10]
rs1816702	5.00×10^{-2}	*$1.95 \times 10^{+0}$*	CT/TT	Re	[49]
rs10495565	N.D.	1.40×10^{-3}	G	Re	[68]
rs1056204	N.D.	1.40×10^{-3}	C	Re	[68]
rs1143634	N.D.	2.70×10^{-2}	C	NR	[27]
rs12469362	N.D.	1.30×10^{-3}	T	Re	[68]
rs4464248	N.D.	1.80×10^{-3}	G	Re	[68]
rs763110	N.D.	1.00×10^{-3}	CC/CT	Re	[21]

NR (allele / genotype frequency higher in nonresponsive patients), Re (allele / genotype frequency higher in responsive patients); N.D. (not defined), ref. (reference); SNPs with more than one independent confirmation are represented in bold; adjusted p-values not originally reported but calculated here are represented in italics

Supplementary Table S3. SNPs linked to long-term anti-TNF response in CD patients.

SNP	p-value	adjusted p-value	allele / genotype association		ref.
rs9904253	9.80×10^{-6}	*$1.93 \times 10^{+0}$*	A	NR	[10]
rs1130864	4.09×10^{-5}	*4.09×10^{-4}*	C	Re	[8]
rs55689715	6.00×10^{-5}	*$1.18 \times 10^{+1}$*	C	NR	[10]
rs2682714	6.10×10^{-5}	*$1.20 \times 10^{+1}$*	C	NR	[10]
rs7201929	8.10×10^{-5}	*$1.59 \times 10^{+1}$*	C	NR	[10]
rs35725751	9.30×10^{-5}	*$1.83 \times 10^{+1}$*	T	NR	[10]
rs9373839	1.13×10^{-3}	*1.13×10^{-2}*	C	Re	[8]
rs12722515	1.90×10^{-3}	*$3.73 \times 10^{+2}$*	A	NR	[10]
rs763110	9.00×10^{-3}	*6.30×10^{-2}*	CC	NR	[47]
rs3814057	9.60×10^{-3}	3.17×10^{-1}	A	Re	[7]
rs11229555	9.90×10^{-3}	*$1.95 \times 10^{+3}$*	T	NR	[10]
rs3748816	1.20×10^{-2}	3.96×10^{-1}	C	Re	[7]
rs6087990	1.30×10^{-2}	*$2.55 \times 10^{+3}$*	C	NR	[10]
rs212388	1.40×10^{-2}	*$2.75 \times 10^{+3}$*	C	NR	[10]
rs3774934	1.40×10^{-2}	*1.40×10^{-1}*	A	Re	[8]
rs1295686	1.60×10^{-2}	5.28×10^{-1}	AA	Re	[7]
rs766748	1.90×10^{-2}	*5.32×10^{-1}*	GG	Re	[86]
rs254560	2.70×10^{-2}	*$5.31 \times 10^{+3}$*	A	NR	[10]
rs4645983	3.00×10^{-2}	9.90×10^{-1}	C	Re	[7]
rs396991	3.10×10^{-2}	$1.02 \times 10^{+0}$	GG+GT	Re	[7]
rs1440088	3.50×10^{-2}	*$6.88 \times 10^{+3}$*	G	NR	[10]
rs12777960	3.70×10^{-2}	$1.22 \times 10^{+0}$	AA+A	Re	[7]
rs9382084	3.70×10^{-2}	*$1.04 \times 10^{+0}$*	GG	Re	[86]
rs2631372	3.90×10^{-2}	$1.29 \times 10^{+0}$	C	Re	[7]
rs1883136	4.10×10^{-2}	*$1.15 \times 10^{+0}$*	CC/CA	Re	[86]
rs2651244	4.10×10^{-2}	*$8.06 \times 10^{+3}$*	A	NR	[10]
rs2476601	4.20×10^{-2}	$1.39 \times 10^{+0}$	AA	Re	[7]
rs9297145	4.60×10^{-2}	*$9.04 \times 10^{+3}$*	C	NR	[10]
rs17119	4.70×10^{-2}	*$9.24 \times 10^{+3}$*	G	NR	[10]
rs12994997	4.80×10^{-2}	*$9.43 \times 10^{+3}$*	G	NR	[10]
rs10210302	4.90×10^{-2}	$1.62 \times 10^{+0}$	CC+CT	Re	[7]
rs1800629	4.90×10^{-2}	*3.43×10^{-1}*	AA+G	NR	[47]
rs194749	4.90×10^{-2}	*$9.63 \times 10^{+3}$*	C	NR	[10]
rs1799724	N.D.	4.00×10^{-2}	CC	Re	[29]

NR (allele / genotype frequency higher in nonresponsive patients), Re (allele / genotype frequency higher in responsive patients); N.D. (not defined), ref. (reference); adjusted p-values not originally reported but calculated here are represented in italics

Supplementary Table S4. Colon mucosa RNA markers linked to anti-TNF response in CD patients.

gene name	NCBI gene ID	log2 FC NR/Re	p-value	adjusted p-value	ref.
ACOD1	730249	1.961	1.31×10^{-4}	4.53×10^{-2}	[12]
ANGPT2	285	1.525	8.47×10^{-5}	3.94×10^{-2}	[12]
AQP9	366	2.437	1.45×10^{-5}	2.37×10^{-2}	[12]
BCL2A1	597	2.080	1.03×10^{-4}	4.08×10^{-2}	[12]
CCL2	6347	1.876	5.68×10^{-5}	3.57×10^{-2}	[12]
CLEC7A	64581	1.539	2.11×10^{-5}	2.61×10^{-2}	[12]
COL12A1	1303	2.118	1.81×10^{-5}	2.38×10^{-2}	[12]
COL15A1	1306	1.620	1.57×10^{-4}	4.83×10^{-2}	[12]
COL7A1	1294	1.623	2.43×10^{-5}	2.72×10^{-2}	[12]
CREB5	9586	1.976	1.33×10^{-4}	4.54×10^{-2}	[12]
CSF3	1440	2.754	1.33×10^{-5}	2.37×10^{-2}	[12]
CSF3R	1441	1.910	1.15×10^{-4}	4.32×10^{-2}	[12]
CSGALNACT1	55790	1.631	5.20×10^{-5}	3.57×10^{-2}	[12]
CTHRC1	115908	1.930	3.49×10^{-5}	3.57×10^{-2}	[12]
CXCL6	6372	2.435	1.16×10^{-7}	6.36×10^{-3}	[12]
CXCR2	3579	1.814	4.69×10^{-5}	3.57×10^{-2}	[12]
EGFL6	25975	1.511	4.92×10^{-5}	3.57×10^{-2}	[12]
ENG	2022	1.799	2.14×10^{-5}	2.61×10^{-2}	[12]
F5	2153	1.957	1.35×10^{-4}	4.55×10^{-2}	[12]
FAM124A	220108	2.176	9.55×10^{-6}	2.37×10^{-2}	[12]
FCGR2A	2212	1.546	1.34×10^{-4}	4.55×10^{-2}	[12]
FCGR2C	9103	1.546	1.34×10^{-4}	4.55×10^{-2}	[12]
FCGR3A	2214	2.107	1.47×10^{-5}	2.37×10^{-2}	[12]
FCGR3B	2215	2.107	1.47×10^{-5}	2.37×10^{-2}	[12]
FCN1	2219	1.632	1.54×10^{-5}	2.38×10^{-2}	[12]
FGF2	2247	1.897	1.74×10^{-5}	2.38×10^{-2}	[12]
FPR1	2357	2.136	4.68×10^{-6}	2.37×10^{-2}	[12]
FPR2	2358	1.711	4.43×10^{-5}	3.57×10^{-2}	[12]
G0S2	50486	1.828	7.36×10^{-6}	2.37×10^{-2}	[12]
HGF	3082	2.362	6.90×10^{-6}	2.37×10^{-2}	[12]
IGFBP5	3488	1.810	2.43×10^{-5}	2.72×10^{-2}	[12]
IL11	3589	3.306	1.41×10^{-5}	2.37×10^{-2}	[12]
		3.184	$<1.00 \times 10^{-2}$	*$<5.00 \times 10^{-2}$*	[30]
IL13RA2	3598	2.761	1.27×10^{-5}	2.37×10^{-2}	[12]
		2.184	$<1.00 \times 10^{-2}$	*$<5.00 \times 10^{-2}$*	[30]
IL1RN	3557	2.044	1.80×10^{-5}	2.38×10^{-2}	[12]
KCNJ15	3772	1.855	8.31×10^{-5}	3.94×10^{-2}	[12]
LILRA1	11024	1.584	7.23×10^{-5}	3.80×10^{-2}	[12]
LILRA2	11027	1.850	1.40×10^{-5}	2.37×10^{-2}	[12]
LILRA6	79168	1.636	2.20×10^{-5}	2.61×10^{-2}	[12]
LILRB2	10288	1.636	2.20×10^{-5}	2.61×10^{-2}	[12]
LILRB3	11025	1.636	2.20×10^{-5}	2.61×10^{-2}	[12]
LOC102725015	102725015	1.636	2.20×10^{-5}	2.61×10^{-2}	[12]
LOC102725029	102725029	1.636	2.20×10^{-5}	2.61×10^{-2}	[12]
LOC102725031	102725031	1.636	2.20×10^{-5}	2.61×10^{-2}	[12]
LOC102725034	102725034	1.636	2.20×10^{-5}	2.61×10^{-2}	[12]
LOC102725035	102725035	1.636	2.20×10^{-5}	2.61×10^{-2}	[12]
LOC645513	645513	-2.192	3.86×10^{-5}	3.57×10^{-2}	[12]
LRRC25	126364	1.827	8.37×10^{-5}	3.94×10^{-2}	[12]
MCEMP1	199675	1.970	2.26×10^{-5}	2.63×10^{-2}	[12]
MGP	4256	1.633	4.38×10^{-5}	3.57×10^{-2}	[12]
MMP1	4312	2.196	3.77×10^{-6}	2.37×10^{-2}	[12]
MMP2	4313	1.762	1.26×10^{-5}	2.37×10^{-2}	[12]
MMP3	4314	2.230	1.24×10^{-4}	4.47×10^{-2}	[12]
MNDA	4332	2.029	1.14×10^{-5}	2.37×10^{-2}	[12]
MUC5AC	4586	3.043	5.40×10^{-5}	3.57×10^{-2}	[12]
NCF2	4688	1.907	9.19×10^{-5}	4.06×10^{-2}	[12]
NRP1	8829	1.939	4.90×10^{-5}	3.57×10^{-2}	[12]
NRP2	8828	1.786	1.19×10^{-4}	4.37×10^{-2}	[12]
OSM	5008	2.731	3.90×10^{-5}	3.57×10^{-2}	[12]
PDLIM4	8572	1.620	1.07×10^{-4}	4.11×10^{-2}	[12]
PI15	51050	2.729	2.52×10^{-5}	2.75×10^{-2}	[12]
PLEK	5341	1.827	1.52×10^{-4}	4.82×10^{-2}	[12]
PLTP	5360	1.660	1.78×10^{-5}	2.38×10^{-2}	[12]
PLXND1	23129	1.856	6.71×10^{-5}	3.63×10^{-2}	[12]
PROK2	60675	3.189	5.79×10^{-6}	2.37×10^{-2}	[12]
PTGS2	5743	2.206	8.55×10^{-5}	3.94×10^{-2}	[12]
		3.059	$<1.00 \times 10^{-2}$	*$<5.00 \times 10^{-2}$*	[30]
PTX3	5806	2.141	1.40×10^{-5}	2.37×10^{-2}	[12]
PYY	5697	-1.760	5.89×10^{-5}	3.57×10^{-2}	[12]
RASSF8	11228	1.713	4.87×10^{-5}	3.57×10^{-2}	[12]
RGS5	8490	1.588	8.57×10^{-5}	3.94×10^{-2}	[12]
S100A12	6283	2.380	3.68×10^{-6}	2.37×10^{-2}	[12]
S100A8	6279	2.491	7.67×10^{-6}	2.37×10^{-2}	[12]
S100A9	6280	2.118	5.61×10^{-5}	3.57×10^{-2}	[12]
SELE	6401	2.232	1.49×10^{-4}	4.80×10^{-2}	[12]
SELL	6402	1.700	1.30×10^{-4}	4.51×10^{-2}	[12]
SERPINB2	5055	2.806	8.52×10^{-6}	2.37×10^{-2}	[12]
SIGLEC5	8778	1.542	1.47×10^{-4}	4.74×10^{-2}	[12]
SLC2A3	6515	1.561	1.54×10^{-4}	4.83×10^{-2}	[12]
STC1	6781	1.514	7.49×10^{-5}	3.89×10^{-2}	[12]
		2.396	$<1.00 \times 10^{-2}$	*$<5.00 \times 10^{-2}$*	[30]
TFAP2A	7020	1.991	4.28×10^{-5}	3.57×10^{-2}	[12]
TFPI2	7980	2.697	1.05×10^{-4}	4.11×10^{-2}	[12]
TMEM71	137835	1.713	5.90×10^{-5}	3.57×10^{-2}	[12]
TNC	3371	1.912	2.74×10^{-5}	2.94×10^{-2}	[12]
TNFAIP6	7130	2.423	4.64×10^{-5}	3.57×10^{-2}	[12]
TNFRSF11B	4982	1.737	$<1.00 \times 10^{-2}$	*$<5.00 \times 10^{-2}$*	[30]
TREM1	54210	2.881	4.81×10^{-5}	3.57×10^{-2}	[12]
TRPM6	140803	-1.587	8.30×10^{-5}	3.94×10^{-2}	[12]
VCAN	1462	1.615	9.99×10^{-5}	4.06×10^{-2}	[12]
VMO1	284013	2.345	8.10×10^{-6}	2.37×10^{-2}	[12]
VNN2	8875	2.126	5.61×10^{-5}	3.57×10^{-2}	[12]

RNA markers with more than one confirmation are represented in bold; adjusted p-values not originally reported but calculated here are represented in italics

Supplementary Table S5. Integrated colon markers linked to short-term anti-TNF response in CD patients.

gene name	NCBI gene ID	SNP	p-value	adjusted p-value	data type	reference
**AC034220.3**	N.A.	rs2522057	2.40×10^{-2}	4.80×10^{-2}	DNA	[28]
		rs35260072	2.40×10^{-2}	4.80×10^{-2}		
		rs1050152	2.40×10^{-2}	7.92×10^{-1}		[7]
		rs2631372	2.60×10^{-2}	8.58×10^{-1}		
ACOD1	730249	N.A.	1.31×10^{-4}	4.53×10^{-2}	RNA	[12]
ACSL6	23305	rs2522057	2.40×10^{-2}	4.80×10^{-2}	DNA	[28]
		rs35260072	2.40×10^{-2}	4.80×10^{-2}		
		rs1050152	2.40×10^{-2}	7.92×10^{-1}		[7]
AMT	275	rs3197999	2.50×10^{-2}	$4.91 \times 10^{+3}$	DNA	[10]
ANGPT2	285	N.A.	8.47×10^{-5}	3.94×10^{-2}	RNA	[12]
AQP9	366	N.A.	1.45×10^{-5}	2.37×10^{-2}	RNA	[12]
BCL2A1	597	N.A.	1.03×10^{-4}	4.08×10^{-2}	RNA	[12]
CASP9	842	rs4645983	N.D.	3.00×10^{-2}	DNA	[21]
			7.20×10^{-3}	2.38×10^{-1}		[7]
CCL2	6347	N.A.	5.68×10^{-5}	3.57×10^{-2}	RNA	[12]
CDC37P1	390688	rs8049439	5.60×10^{-3}	1.85×10^{-1}	DNA	[7]
CLEC7A	64581	N.A.	2.11×10^{-5}	2.61×10^{-2}	RNA	[12]
COL12A1	1303	N.A.	1.81×10^{-5}	2.38×10^{-2}	RNA	[12]
COL15A1	1306	N.A.	1.57×10^{-4}	4.83×10^{-2}	RNA	[12]
COL7A1	1294	N.A.	2.43×10^{-5}	2.72×10^{-2}	RNA	[12]
CREB5	9586	N.A.	1.33×10^{-4}	4.54×10^{-2}	RNA	[12]
CSF3	1440	N.A.	1.33×10^{-5}	2.37×10^{-2}	RNA	[12]
CSF3R	1441	N.A.	1.15×10^{-4}	4.32×10^{-2}	RNA	[12]
CSGALNACT1	55790	N.A.	5.20×10^{-5}	3.57×10^{-2}	RNA	[12]
CTHRC1	115908	N.A.	3.49×10^{-5}	3.57×10^{-2}	RNA	[12]
CXCL6	6372	N.A.	1.16×10^{-7}	6.36×10^{-3}	RNA	[12]
CXCR2	3579	N.A.	4.69×10^{-5}	3.57×10^{-2}	RNA	[12]
EGFL6	25975	N.A.	4.92×10^{-5}	3.57×10^{-2}	RNA	[12]
ENG	2022	N.A.	2.14×10^{-5}	2.61×10^{-2}	RNA	[12]
F5	2153	N.A.	1.35×10^{-4}	4.55×10^{-2}	RNA	[12]
FAM124A	220108	N.A.	9.55×10^{-6}	2.37×10^{-2}	RNA	[12]
FAS	355	rs1800682	3.50×10^{-2}	$1.16 \times 10^{+0}$	DNA	[7]
FCGR2A	2212	N.A.	1.34×10^{-4}	4.55×10^{-2}	RNA	[12]
FCGR2C	9103	rs396991	3.00×10^{-3}	3.00×10^{-3}	DNA	[23]
		N.A.	1.34×10^{-4}	4.55×10^{-2}	RNA	[12]
		rs396991	2.10×10^{-2}	6.93×10^{-1}	DNA	[7]
FCGR3A	2214	N.A.	1.47×10^{-5}	2.37×10^{-2}	RNA	[12]
FCGR3B	2215	N.A.	1.47×10^{-5}	2.37×10^{-2}	RNA	[12]
FCN1	2219	N.A.	1.54×10^{-5}	2.38×10^{-2}	RNA	[12]
FGF2	2247	N.A.	1.74×10^{-5}	2.38×10^{-2}	RNA	[12]
FPR1	2357	N.A.	4.68×10^{-6}	2.37×10^{-2}	RNA	[12]
FPR2	2358	N.A.	4.43×10^{-5}	3.57×10^{-2}	RNA	[12]
G0S2	50486	N.A.	7.36×10^{-6}	2.37×10^{-2}	RNA	[12]
GNA12	2768	rs1182188	1.40×10^{-2}	$2.75 \times 10+3$	DNA	[10]
GSDMA	284110	rs2872507	3.60×10^{-2}	$1.19 \times 10^{+0}$	DNA	[7]

GSDMB	55876	rs2872507	3.60×10^{-2}	$1.19 \times 10^{+0}$	DNA	[7]
HFE	3077	rs2071303	8.20×10^{-3}	1.64×10^{-2}	DNA	[9]
HGF	3082	N.A.	6.90×10^{-6}	2.37×10^{-2}	RNA	[12]
HSPA7	3311	rs396991	3.00×10^{-3}	3.00×10^{-3}	DNA	[23]
			2.10×10^{-2}	6.93×10^{-1}		[7]
IGFBP5	3488	N.A.	2.43×10^{-5}	2.72×10^{-2}	RNA	[12]
IL11	3589	N.A.	1.41×10^{-5}	2.37×10^{-2}	RNA	[12]
			$<1.00 \times 10^{-2}$	$<5.00 \times 10^{-2}$		[30]
IL12RB1	3594	rs11575934	2.20×10^{-2}	7.26×10^{-1}	DNA	[7]
IL13RA2	3598	N.A.	1.27×10^{-5}	2.37×10^{-2}	RNA	[12]
			$<1.00 \times 10^{-2}$	$<5.00 \times 10^{-2}$		[30]
IL1RN	3557	N.A.	1.80×10^{-5}	2.38×10^{-2}	RNA	[12]
ITIH4	3700	rs9847710	1.30×10^{-2}	$2.55 \times 10^{+3}$	DNA	[10]
KCNJ15	3772	N.A.	8.31×10^{-5}	3.94×10^{-2}	RNA	[12]
KIF3A	11127	rs2070874	3.60×10^{-2}	$1.19 \times 10^{+0}$	DNA	[7]
LILRA1	11024	N.A.	7.23×10^{-5}	3.80×10^{-2}	RNA	[12]
LILRA2	11027	N.A.	1.40×10^{-5}	2.37×10^{-2}	RNA	[12]
LILRA6	79168	N.A.	2.20×10^{-5}	2.61×10^{-2}	RNA	[12]
LILRB2	10288	N.A.	2.20×10^{-5}	2.61×10^{-2}	RNA	[12]
LILRB3	11025	N.A.	2.20×10^{-5}	2.61×10^{-2}	RNA	[12]
LOC102725015	102725015	N.A.	2.20×10^{-5}	2.61×10^{-2}	RNA	[12]
LOC102725029	102725029	N.A.	2.20×10^{-5}	2.61×10^{-2}	RNA	[12]
LOC102725031	102725031	N.A.	2.20×10^{-5}	2.61×10^{-2}	RNA	[12]
LOC102725034	102725034	N.A.	2.20×10^{-5}	2.61×10^{-2}	RNA	[12]
LOC102725035	102725035	N.A.	2.20×10^{-5}	2.61×10^{-2}	RNA	[12]
LOC645513	645513	N.A.	3.86×10^{-5}	3.57×10^{-2}	RNA	[12]
LRRC25	126364	N.A.	8.37×10^{-5}	3.94×10^{-2}	RNA	[12]
MCEMP1	199675	N.A.	2.26×10^{-5}	2.63×10^{-2}	RNA	[12]
MGP	4256	N.A.	4.38×10^{-5}	3.57×10^{-2}	RNA	[12]
MMEL1	79258	rs3748816	1.20×10^{-2}	3.96×10^{-1}	DNA	[7]
MMP1	4312	N.A.	3.77×10^{-6}	2.37×10^{-2}	RNA	[12]
MMP2	4313	N.A.	1.26×10^{-5}	2.37×10^{-2}	RNA	[12]
MMP3	4314	N.A.	1.24×10^{-4}	4.47×10^{-2}	RNA	[12]
MNDA	4332	N.A.	1.14×10^{-5}	2.37×10^{-2}	RNA	[12]
MUC5AC	4586	N.A.	5.40×10^{-5}	3.57×10^{-2}	RNA	[12]
NCF2	4688	N.A.	9.19×10^{-5}	4.06×10^{-2}	RNA	[12]
NPIPB7	440350	rs8049439	5.60×10^{-3}	1.85×10^{-1}	DNA	[7]
NRP1	8829	N.A.	4.90×10^{-5}	3.57×10^{-2}	RNA	[12]
NRP2	8828	N.A.	1.19×10^{-4}	4.37×10^{-2}	RNA	[12]
OSM	5008	N.A.	3.90×10^{-5}	3.57×10^{-2}	RNA	[12]
PARK7	11315	rs3766606	2.20×10^{-2}	$4.32 \times 10^{+3}$	DNA	[10]
PDLIM4	8572	N.A.	1.07×10^{-4}	4.11×10^{-2}	RNA	[12]
PGAP3	93210	rs2872507	3.60×10^{-2}	$1.19 \times 10^{+0}$	DNA	[7]
PI15	51050	N.A.	2.52×10^{-5}	2.75×10^{-2}	RNA	[12]
PLEK	5341	N.A.	1.52×10^{-4}	4.82×10^{-2}	RNA	[12]
PLTP	5360	N.A.	1.78×10^{-5}	2.38×10^{-2}	RNA	[12]
PLXND1	23129	N.A.	6.71×10^{-5}	3.63×10^{-2}	RNA	[12]
PROK2	60675	N.A.	5.79×10^{-6}	2.37×10^{-2}	RNA	[12]
PTGS2	5743	N.A.	8.55×10^{-5}	3.94×10^{-2}	RNA	[12]
			$<1.00 \times 10^{-2}$	$<5.00 \times 10^{-2}$		[30]

PTX3	5806	N.A.	1.40×10^{-5}	2.37×10^{-2}	RNA	[12]
PYY	5697	N.A.	5.89×10^{-5}	3.57×10^{-2}	RNA	[12]
RASSF8	11228	N.A.	4.87×10^{-5}	3.57×10^{-2}	RNA	[12]
RBM6	10180	rs3197999	2.50×10^{-2}	*$4.91 \times 10^{+3}$*	DNA	[10]
RFT1	91869	rs9847710	1.30×10^{-2}	*$2.55 \times 10^{+3}$*	DNA	[10]
RGS5	8490	N.A.	8.57×10^{-5}	3.94×10^{-2}	RNA	[12]
RNF123	63891	rs3197999	2.50×10^{-2}	*$4.91 \times 10^{+3}$*	DNA	[10]
RP11-1348G14.4	N.A.	rs8049439	5.60×10^{-3}	1.85×10^{-1}	DNA	[7]
S100A12	6283	N.A.	3.68×10^{-6}	2.37×10^{-2}	RNA	[12]
S100A8	6279	N.A.	7.67×10^{-6}	2.37×10^{-2}	RNA	[12]
			$<5.00 \times 10^{-3}$	N.D.	protein	[38]
			$<5.00 \times 10^{-3}$	N.D.		[39]
			3.00×10^{-2}	N.D.		[37]
S100A9	6280	N.A.	5.61×10^{-5}	3.57×10^{-2}	RNA	[12]
			$<5.00 \times 10^{-3}$	N.D.	protein	[38]
			$<5.00 \times 10^{-3}$	N.D.		[39]
			3.00×10^{-2}	N.D.		[37]
SELE	6401	N.A.	1.49×10^{-4}	4.80×10^{-2}	RNA	[12]
SELL	6402	N.A.	1.30×10^{-4}	4.51×10^{-2}	RNA	[12]
SERPINB2	5055	N.A.	8.52×10^{-6}	2.37×10^{-2}	RNA	[12]
SFMBT1	51460	rs9847710	1.30×10^{-2}	*$2.55 \times 10^{+3}$*	DNA	[10]
SIGLEC5	8778	N.A.	1.47×10^{-4}	4.74×10^{-2}	RNA	[12]
SLC22A4	6583	rs35260072	2.40×10^{-2}	*4.80×10^{-2}*	DNA	[28]
		rs1050152	2.40×10^{-2}	7.92×10^{-1}		[7]
SLC22A5	6584	rs35260072	2.40×10^{-2}	*4.80×10^{-2}*	DNA	[28]
		rs1050152	2.40×10^{-2}	7.92×10^{-1}		[7]
		rs2631372	2.60×10^{-2}	8.58×10^{-1}		
SLC2A3	6515	N.A.	1.54×10^{-4}	4.83×10^{-2}	RNA	[12]
STC1	6781	N.A.	7.49×10^{-5}	3.89×10^{-2}	RNA	[12]
			$<1.00 \times 10^{-2}$	$<5.00 \times 10^{-2}$		[30]
SULT1A1	6817	rs8049439	5.60×10^{-3}	1.85×10^{-1}	DNA	[7]
TARS2	80222	rs13294	4.50×10^{-2}	*$1.49 \times 10^{+0}$*	DNA	[7]
TFAP2A	7020	N.A.	4.28×10^{-5}	3.57×10^{-2}	RNA	[12]
TFPI2	7980	N.A.	1.05×10^{-4}	4.11×10^{-2}	RNA	[12]
TMEM71	137835	N.A.	5.90×10^{-5}	3.57×10^{-2}	RNA	[12]
TNC	3371	N.A.	2.74×10^{-5}	2.94×10^{-2}	RNA	[12]
TNF	7124	N.A.	4.00×10^{-4}	N.D.	protein	[40]
TNFAIP6	7130	N.A.	4.64×10^{-5}	3.57×10^{-2}	RNA	[12]
TNFRSF11B	4982	N.A.	$<1.00 \times 10^{-2}$	$<5.00 \times 10^{-2}$	RNA	[30]
TREM1	54210	N.A.	4.81×10^{-5}	3.57×10^{-2}	RNA	[12]
TRPM6	140803	N.A.	8.30×10^{-5}	3.94×10^{-2}	RNA	[12]
TUFM	7284	rs8049439	5.60×10^{-3}	1.85×10^{-1}	DNA	[7]
UBA7	7318	rs3197999	2.50×10^{-2}	*$4.91 \times 10^{+3}$*	DNA	[10]
VCAN	1462	N.A.	9.99×10^{-5}	4.06×10^{-2}	RNA	[12]
VMO1	284013	N.A.	8.10×10^{-6}	2.37×10^{-2}	RNA	[12]
VNN2	8875	N.A.	5.61×10^{-5}	3.57×10^{-2}	RNA	[12]

Markers with more than one confirmation are represented in bold; adjusted p-values not originally reported but calculated here are represented in italics; N.D. (not defined); N.A. (not applicable)

Supplementary Table S6. Integrated colon markers linked to long-term anti-TNF response in CD patients.

gene name	NCBI gene ID	SNP	p-value	adjusted p-value	data type	reference
AC034220.3	N.A.	rs2631372	3.90×10^{-2}	*$1.29 \times 10^{+0}$*	DNA	[7]
ATP6V1G2	534	rs1799724	2.50×10^{-3}	4.00×10^{-2}	DNA	[29]
C4A	720	rs1800629	4.90×10^{-2}	*3.43×10^{-1}*	DNA	[47]
CASP9	842	rs4645983	3.00×10^{-2}	9.90×10^{-1}	DNA	[7]
CCHCR1	54535	rs1800629	4.90×10^{-2}	*3.43×10^{-1}*	DNA	[47]
		rs1799724	2.50×10^{-3}	4.00×10^{-2}		[29]
CDC37P1	390688	rs7201929	8.10×10^{-5}	*$1.59 \times 10^{+1}$*	DNA	[10]
		rs35725751	9.30×10^{-5}	*$1.83 \times 10^{+1}$*		
COMMD7	149951	rs6087990	1.30×10^{-2}	*$2.55 \times 10^{+3}$*	DNA	[10]
CYP21A1P	1590	rs1800629	4.90×10^{-2}	*3.43×10^{-1}*	DNA	[47]
DNMT3B	1789	rs6087990	1.30×10^{-2}	*$2.55 \times 10^{+3}$*	DNA	[10]
FCGR2C	9103	rs396991	3.10×10^{-2}	$1.02 \times 10^{+0}$	DNA	[7]
HLA-C	3107	rs1800629	4.90×10^{-2}	*3.43×10^{-1}*	DNA	[47]
HSPA7	3311	rs396991	3.10×10^{-2}	$1.02 \times 10^{+0}$	DNA	[7]
MICA	100507436	rs1799724	2.50×10^{-3}	4.00×10^{-2}	DNA	[29]
MMEL1	79258	rs3748816	1.20×10^{-2}	3.96×10^{-1}	DNA	[7]
PSORS1C1	170679	rs1800629	4.90×10^{-2}	*3.43×10^{-1}*	DNA	[47]
SLC22A5	6584	rs2631372	3.90×10^{-2}	$1.29 \times 10^{+0}$	DNA	[7]
TRAF3IP2-AS1	643749	rs1883136	4.10×10^{-2}	*$1.15 \times 10^{+0}$*	DNA	[86]
TUFM	7284	rs7201929	8.10×10^{-5}	*$1.59 \times 10^{+1}$*	DNA	[10]
		rs35725751	9.30×10^{-5}	*$1.83 \times 10^{+1}$*		

Markers with more than one confirmation are represented in bold; adjusted p-values not originally reported but calculated here are represented in italics; N.A. (not applicable)

Supplementary Table S7. Integrated blood markers linked to short-term anti-TNF response in CD patients.

gene name	NCBI gene ID	SNP	p-value	adjusted p-value	data type	reference
AC007278.2	N.A.	rs6708413	4.50×10^{-2}	*$8.84 \times 10^{+3}$*	DNA	[10]
AC007278.3	N.A.	rs6708413	4.50×10^{-2}	*$8.84 \times 10^{+3}$*	DNA	[10]
AC034220.3	N.A.	rs2631372	2.60×10^{-2}	8.58×10^{-1}	DNA	[7]
**AC116366.6**	N.A.	rs2522057	2.40×10^{-2}	*4.80×10^{-2}*	DNA	[28]
		rs35260072	2.40×10^{-2}	*4.80×10^{-2}*		
		rs1050152	2.40×10^{-2}	7.92×10^{-1}		[7]
		rs2631372	2.60×10^{-2}	8.58×10^{-1}		
ACTA2	59	rs1800682	3.50×10^{-2}	$1.16 \times 10^{+0}$	DNA	[7]
CREM	1390	rs12777960	6.70×10^{-3}	2.21×10^{-1}	DNA	[7]
CRP	1401	N.A.	2.00×10^{-2}	N.D.	protein	[34]
			7.00×10^{-4}	N.D.		[35]
			1.50×10^{-2}	N.D.		[36]
			4.00×10^{-2}	N.D.		[37]
CXCL8	3576	N.A.	1.00×10^{-2}	N.D.		[41]
DEFA1	1667	N.A.	6.72×10^{-3}	9.13×10^{-1}	RNA	[32]
DEFA1B	728358	N.A.	6.84×10^{-3}	9.13×10^{-1}	RNA	[32]
DEFA3	1668	N.A.	6.72×10^{-3}	9.13×10^{-1}	RNA	[32]
FAS	355	rs1800682	3.50×10^{-2}	$1.16 \times 10^{+0}$	DNA	[7]
GNA12	2768	rs1182188	1.40×10^{-2}	*$2.75 \times 10^{+3}$*	DNA	[10]
GSDMB	55876	rs2872507	3.60×10^{-2}	$1.19 \times 10^{+0}$	DNA	[7]
IL12RB1	3594	rs11575934	2.20×10^{-2}	7.26×10^{-1}	DNA	[7]
IL15	3600	N.A.	1.00×10^{-2}	N.D.	protein	[42]
IL17A	3605	N.A.	4.00×10^{-2}	N.D.	protein	[43]
IL18RAP	8807	rs6708413	4.50×10^{-2}	*$8.84 \times 10^{+3}$*	DNA	[10]
ITIH4	3700	rs9847710	1.30×10^{-2}	*$2.55 \times 10^{+3}$*	DNA	[10]
LTBR	4055	rs4149570	4.00×10^{-2}	*$1.56 \times 10^{+0}$*	DNA	[49]
MMEL1	79258	rs3748816	1.20×10^{-2}	3.96×10^{-1}	DNA	[7]
MUSTN1	389125	rs9847710	1.30×10^{-2}	*$2.55 \times 10^{+3}$*	DNA	[10]
NPIPB7	440350	rs8049439	5.60×10^{-3}	1.85×10^{-1}	DNA	[7]
NT5DC2	64943	rs9847710	1.30×10^{-2}	*$2.55 \times 10^{+3}$*	DNA	[10]
ORMDL3	94103	rs2872507	3.60×10^{-2}	$1.19 \times 10^{+0}$	DNA	[7]
PARK7	11315	rs3766606	2.20×10^{-2}	*$4.32 \times 10^{+3}$*	DNA	[10]
PRXL2B	127281	rs3748816	1.20×10^{-2}	3.96×10^{-1}	DNA	[7]
RBM43	375287	rs11677200	1.70×10^{-2}	*2.04×10^{-1}*	DNA	[85]
RBM6	10180	rs3197999	2.50×10^{-2}	*$4.91 \times 10^{+3}$*	DNA	[10]
RPRD2	23248	rs13294	4.50×10^{-2}	$1.49 \times 10^{+0}$	DNA	[7]
RPS23P10	100419471	rs396991	3.00×10^{-3}	3.00×10^{-3}	DNA	[23]
			2.10×10^{-2}	6.93×10^{-1}		[7]
SCARNA4	677771	N.A.	2.24×10^{-2}	8.96×10^{-1}	RNA	[31]
SFG29	112869	rs8049439	5.60×10^{-3}	1.85×10^{-1}	DNA	[7]
SLC22A5	6584	rs2522057	2.40×10^{-2}	*4.80×10^{-2}*	DNA	[28]
		rs35260072	2.40×10^{-2}	*4.80×10^{-2}*		
		rs1050152	2.40×10^{-2}	7.92×10^{-1}		[7]
SPNS1	83985	rs8049439	5.60×10^{-3}	1.85×10^{-1}	DNA	[7]
SULT1A1	6817	rs8049439	5.60×10^{-3}	1.85×10^{-1}	DNA	[7]
SULT1A2	6799	rs8049439	5.60×10^{-3}	1.85×10^{-1}	DNA	[7]
TGFB1	7040	N.A.	$<5.00 \times 10^{-3}$	N.D.	protein	[44]
TLR4	7099	rs5030728	3.00×10^{-2}	*$1.17 \times 10^{+0}$*	DNA	[49]
TMEM176A	55365	N.A.	2.36×10^{-2}	8.96×10^{-1}	RNA	[31]
TNF	7124	N.A.	3.50×10^{-2}	N.D.	protein	[45]

TNFAIP6	7130	rs11677200	1.70×10^{-2}	*2.04×10^{-1}*	DNA	[85]
TNFSF15	9966	rs4263839	2.70×10^{-2}	8.91×10^{-1}	DNA	[7]
TRIM38	10475	rs2071303	8.20×10^{-3}	*1.64×10^{-2}*	DNA	[9]
TUFM	7284	rs8049439	5.60×10^{-3}	1.85×10^{-1}	DNA	[7]
UBA7	7318	rs3197999	2.50×10^{-2}	*$4.91 \times 10^{+3}$*	DNA	[10]
ZFP90	146198	rs1728785	4.50×10^{-2}	*$8.84 \times 10^{+3}$*	DNA	[10]

Markers with more than one confirmation are represented in bold; adjusted p-values not originally reported but calculated here are represented in italics; N.A. (not applicable); N.D. (not defined)

Supplementary Table S8. Integrated blood markers linked to long-term anti-TNF response in CD patients.

gene name	NCBI gene ID	SNP	p-value	adjusted p-value	data type	reference
AC034220.3	N.A.	rs2631372	3.90×10^{-2}	$1.29 \times$ *10^{+0}*	DNA	[7]
AC116366.6	N.A.	rs2631372	3.90×10^{-2}	$1.29 \times$ *10^{+0}*	DNA	[7]
ATP6V1G2	534	rs1799724	2.50×10^{-3}	4.00×10^{-2}	DNA	[29]
C4A	720	rs1800629	4.90×10^{-2}	*3.43×10^{-1}*	DNA	[47]
C4B	721	rs1800629	4.90×10^{-2}	*3.43×10^{-1}*	DNA	[47]
CCHCR1	54535	rs1800629	4.90×10^{-2}	*3.43×10^{-1}*	DNA	[47]
CREM	1390	rs12777960	3.70×10^{-2}	$1.22 \times$ *10^{+0}*	DNA	[7]
CYP21A1P	1590	rs1800629	4.90×10^{-2}	*3.43×10^{-1}*	DNA	[47]
CYP21A2	1589	rs1800629	4.90×10^{-2}	*3.43×10^{-1}*	DNA	[47]
IL6	3569	N.A.	2.90×10^{-2}	N.D.	protein	[46]
LINC00243	401247	rs1800629	4.90×10^{-2}	*3.43×10^{-1}*	DNA	[47]
MAPRE1	22919	rs6087990	1.30×10^{-2}	*$2.55 \times 10^{+3}$*	DNA	[10]
MMEL1	79258	rs3748816	1.20×10^{-2}	3.96×10^{-1}	DNA	[7]
NCR3	259197	rs1799724	2.50×10^{-3}	4.00×10^{-2}	DNA	[29]
POU5F1	5460	rs1800629	4.90×10^{-2}	*3.43×10^{-1}*	DNA	[47]
PRXL2B	127281	rs3748816	1.20×10^{-2}	3.96×10^{-1}	DNA	[7]
RP5-1085F17.3	N.A.	rs6087990	1.30×10^{-2}	*$2.55 \times 10^{+3}$*	DNA	[10]
RPS23P10	100419471	rs396991	3.10×10^{-2}	$1.02 \times$ *10^{+0}*	DNA	[7]
SULT1A2	6799	rs7201929	8.10×10^{-5}	*$1.59 \times 10^{+1}$*	DNA	[10]
		rs35725751	9.30×10^{-5}	*$1.83 \times 10^{+1}$*		
TREM1	54210	N.A.	2.00×10^{-2}	*2.00×10^{-2}*	RNA	[33]
			1.00×10^{-3}	N.D.	protein	
TUFM	7284	rs7201929	8.10×10^{-5}	*$1.59 \times 10^{+1}$*	DNA	[10]
		rs35725751	9.30×10^{-5}	*$1.83 \times 10^{+1}$*		

Markers with more than one confirmation are represented in bold; adjusted p-values not originally reported but calculated here are represented in italics; N.A. (not applicable); N.D. (not defined)

Supplementary Table S9. GO analysis of the integrated colon markers linked to the short-term anti-TNF response in CD patients.

GO ID	GO term	ontology source	p-value	adjusted p-value	% assoc. genes	nr. assoc. genes
GO:0005125	cytokine activity	MF	8.48×10^{-7}	5.43×10^{-5}	9.33	7
GO:0006935	chemotaxis	BP	2.75×10^{-6}	1.73×10^{-4}	4.06	11
GO:0048018	receptor ligand activity	MF	2.86×10^{-6}	1.77×10^{-4}	5.26	9
GO:0033138	positive regulation of peptidyl-serine phosphorylation	BP	6.35×10^{-6}	3.87×10^{-4}	6.93	7
GO:0002683	negative regulation of immune system process	BP	1.42×10^{-5}	8.37×10^{-4}	3.80	10
GO:0050900	leukocyte migration	BP	1.40×10^{-5}	8.42×10^{-4}	4.33	9
GO:0032102	negative regulation of response to external stimulus	BP	1.74×10^{-5}	1.01×10^{-3}	4.91	8
GO:0060326	cell chemotaxis	BP	2.35×10^{-5}	1.34×10^{-3}	4.05	9
GO:0050922	negative regulation of chemotaxis	BP	4.55×10^{-5}	2.45×10^{-3}	13.79	4
GO:0050731	positive regulation of peptidyl-tyrosine phosphorylation	BP	4.42×10^{-5}	2.47×10^{-3}	5.15	7
GO:0019730	antimicrobial humoral response	BP	4.51×10^{-5}	2.48×10^{-3}	6.45	6
GO:0042056	chemoattractant activity	MF	6.77×10^{-5}	3.59×10^{-3}	12.50	4
GO:0002768	immune response-regulating cell surface receptor signaling pathway	BP	7.57×10^{-5}	3.94×10^{-3}	5.88	6
GO:0007159	leukocyte cell-cell adhesion	BP	9.86×10^{-5}	5.03×10^{-3}	3.85	8
GO:0071222	cellular response to lipopolysaccharide	BP	1.40×10^{-4}	7.01×10^{-3}	5.26	6
GO:0097529	myeloid leukocyte migration	BP	1.86×10^{-4}	9.10×10^{-3}	5.00	6
GO:0006909	phagocytosis	BP	1.94×10^{-4}	9.31×10^{-3}	6.49	5
GO:0030336	negative regulation of cell migration	BP	2.15×10^{-4}	1.01×10^{-2}	3.43	8
GO:0032103	positive regulation of response to external stimulus	BP	2.30×10^{-4}	1.06×10^{-2}	3.95	7
GO:0006954	inflammatory response	BP	2.55×10^{-4}	1.15×10^{-2}	3.89	7
GO:0031663	lipopolysaccharide-mediated signaling pathway	BP	2.62×10^{-4}	1.15×10^{-2}	8.89	4
GO:0030593	neutrophil chemotaxis	BP	2.85×10^{-4}	1.23×10^{-2}	8.70	4
GO:0001516	prostaglandin biosynthetic process	BP	3.40×10^{-4}	1.43×10^{-2}	15.00	3
GO:0048246	macrophage chemotaxis	BP	3.40×10^{-4}	1.43×10^{-2}	15.00	3
GO:0048661	positive regulation of smooth muscle cell proliferation	BP	3.65×10^{-4}	1.49×10^{-2}	8.16	4
GO:0050901	leukocyte tethering or rolling	BP	3.94×10^{-4}	1.58×10^{-2}	14.29	3
GO:0006897	endocytosis	BP	4.27×10^{-4}	1.67×10^{-2}	3.10	8
GO:0050920	regulation of chemotaxis	BP	4.43×10^{-4}	1.68×10^{-2}	4.26	6
GO:0042119	neutrophil activation	BP	5.20×10^{-4}	1.87×10^{-2}	13.04	3
GO:0030595	leukocyte chemotaxis	BP	5.14×10^{-4}	1.90×10^{-2}	4.14	6
GO:0045670	regulation of osteoclast differentiation	BP	5.91×10^{-4}	2.07×10^{-2}	12.50	3
GO:0051897	positive regulation of protein kinase B signaling	BP	2.13×10^{-2}	2.13×10^{-2}	3.53	3
GO:0030888	regulation of B cell proliferation	BP	7.52×10^{-4}	2.56×10^{-2}	11.54	3
GO:2001234	negative regulation of apoptotic signaling pathway	BP	9.50×10^{-4}	3.04×10^{-2}	3.68	6
GO:0061138	morphogenesis of a branching epithelium	BP	9.38×10^{-4}	3.09×10^{-2}	10.71	3
GO:1901031	regulation of response to reactive oxygen species	BP	1.04×10^{-3}	3.23×10^{-2}	10.34	3
GO:0050715	positive regulation of cytokine secretion	BP	1.18×10^{-3}	3.53×10^{-2}	4.39	5
GO:0045807	positive regulation of endocytosis	BP	1.87×10^{-2}	3.75×10^{-2}	3.70	3
GO:0010596	negative regulation of endothelial cell migration	BP	9.96×10^{-3}	3.98×10^{-2}	4.69	3
GO:0008083	growth factor activity	MF	1.52×10^{-3}	4.42×10^{-2}	9.09	3
GO:0050670	regulation of lymphocyte proliferation	BP	1.90×10^{-3}	4.56×10^{-2}	3.94	5

GO:0002822	regulation of adaptive immune response based on somatic recombination of immune receptors built from immunoglobulin superfamily domains	BP	9.13×10^{-3}	4.57×10^{-2}	4.84	3
GO:0006898	receptor-mediated endocytosis	BP	1.84×10^{-3}	4.59×10^{-2}	3.97	5
GO:0006631	fatty acid metabolic process	BP	1.72×10^{-3}	4.65×10^{-2}	3.28	6
GO:0035987	endodermal cell differentiation	BP	1.66×10^{-3}	4.65×10^{-2}	8.82	3
GO:0009790	embryo development	BP	1.82×10^{-3}	4.73×10^{-2}	3.24	6
GO:0043281	regulation of cysteine-type endopeptidase activity involved in apoptotic process	BP	1.82×10^{-3}	4.73×10^{-2}	3.24	6

MF (molecular function), BP (biological process)

Supplementary Table S10. GO analysis of the extended interactome of integrated colon markers linked to the short-term anti-TNF response in CD patients.

GO ID	GO term	ontology source	p-value	adjusted p-value	% assoc. genes	nr. assoc. genes
GO:0006915	apoptotic process	BP	1.62×10^{-26}	1.84×10^{-23}	8.19	92
GO:0048583	regulation of response to stimulus	BP	6.61×10^{-25}	7.52×10^{-22}	5.80	135
GO:0048584	positive regulation of response to stimulus	BP	1.38×10^{-24}	1.57×10^{-21}	7.20	100
GO:0007166	cell surface receptor signaling pathway	BP	6.43×10^{-23}	7.30×10^{-20}	7.25	93
GO:0042981	regulation of apoptotic process	BP	7.07×10^{-23}	8.02×10^{-20}	8.28	79
GO:0007165	signal transduction	BP	7.47×10^{-23}	8.46×10^{-20}	5.32	143
GO:0010941	regulation of cell death	BP	5.32×10^{-22}	6.02×10^{-19}	7.70	83
GO:0070887	cellular response to chemical stimulus	BP	4.68×10^{-21}	5.29×10^{-18}	6.25	105
GO:0048518	positive regulation of biological process	BP	9.53×10^{-21}	1.08×10^{-17}	4.44	179
GO:0051246	regulation of protein metabolic process	BP	3.68×10^{-20}	4.16×10^{-17}	5.89	110
GO:0032268	regulation of cellular protein metabolic process	BP	1.03×10^{-19}	1.16×10^{-16}	6.00	105
GO:0009966	regulation of signal transduction	BP	3.47×10^{-19}	3.91×10^{-16}	5.87	106
GO:0048519	negative regulation of biological process	BP	4.38×10^{-19}	4.93×10^{-16}	4.61	157
GO:0010646	regulation of cell communication	BP	1.00×10^{-18}	1.13×10^{-15}	5.71	108
GO:0071310	cellular response to organic substance	BP	1.81×10^{-18}	2.04×10^{-15}	6.66	85
GO:0023051	regulation of signaling	BP	2.20×10^{-18}	2.47×10^{-15}	5.65	108
GO:0010033	response to organic substance	BP	2.43×10^{-18}	2.73×10^{-15}	6.14	95
GO:0048523	negative regulation of cellular process	BP	3.54×10^{-18}	3.97×10^{-15}	4.72	145
GO:0019899	enzyme binding	MF	4.03×10^{-18}	4.51×10^{-15}	5.82	102
GO:0048522	positive regulation of cellular process	BP	5.46×10^{-18}	6.11×10^{-15}	4.44	161
GO:0097190	apoptotic signaling pathway	BP	9.74×10^{-18}	1.09×10^{-14}	10.77	46
GO:0023056	positive regulation of signaling	BP	1.05×10^{-17}	1.18×10^{-14}	7.08	75
GO:0031399	regulation of protein modification process	BP	1.12×10^{-17}	1.25×10^{-14}	6.52	84
GO:0032270	positive regulation of cellular protein metabolic process	BP	1.33×10^{-17}	1.48×10^{-14}	6.85	78
GO:0051247	positive regulation of protein metabolic process	BP	1.46×10^{-17}	1.62×10^{-14}	6.66	81
GO:0010647	positive regulation of cell communication	BP	2.53×10^{-17}	2.82×10^{-14}	7.03	74
GO:0009967	positive regulation of signal transduction	BP	3.88×10^{-17}	4.31×10^{-14}	7.12	72
GO:0035556	intracellular signal transduction	BP	4.46×10^{-17}	4.96×10^{-14}	5.92	94
GO:0080134	regulation of response to stress	BP	1.26×10^{-16}	1.40×10^{-13}	7.35	67
GO:0051128	regulation of cellular component organization	BP	6.85×10^{-16}	7.60×10^{-13}	5.83	90
GO:2001233	regulation of apoptotic signaling pathway	BP	7.24×10^{-16}	8.02×10^{-13}	12.29	36
GO:0043065	positive regulation of apoptotic process	BP	1.13×10^{-15}	1.26×10^{-12}	10.14	43
GO:0043066	negative regulation of apoptotic process	BP	1.20×10^{-15}	1.33×10^{-12}	9.16	48
GO:0044267	cellular protein metabolic process	BP	1.82×10^{-15}	2.01×10^{-12}	4.54	137
GO:0031401	positive regulation of protein modification process	BP	1.84×10^{-15}	2.03×10^{-12}	7.20	64
GO:0010942	positive regulation of cell death	BP	2.00×10^{-15}	2.21×10^{-12}	9.57	45
GO:0019538	protein metabolic process	BP	2.11×10^{-15}	2.32×10^{-12}	4.39	146
GO:1902531	regulation of intracellular signal transduction	BP	2.50×10^{-15}	2.75×10^{-12}	6.40	75
GO:0060548	negative regulation of cell death	BP	2.62×10^{-15}	2.88×10^{-12}	8.53	51
GO:0031625	ubiquitin protein ligase binding	MF	6.81×10^{-15}	7.48×10^{-12}	11.78	35
GO:0050794	regulation of cellular process	BP	6.86×10^{-15}	7.53×10^{-12}	3.61	215
GO:0030162	regulation of proteolysis	BP	8.03×10^{-15}	8.81×10^{-12}	9.22	45
GO:0006464	cellular protein modification process	BP	8.81×10^{-15}	9.65×10^{-12}	4.81	118
GO:0051173	positive regulation of nitrogen compound metabolic	BP	1.46×10^{-14}	1.60×10^{-11}	4.91	112
GO:0042325	regulation of phosphorylation	BP	1.76×10^{-14}	1.92×10^{-11}	6.46	70
GO:0006468	protein phosphorylation	BP	1.89×10^{-14}	2.07×10^{-11}	6.01	78
GO:0031325	positive regulation of cellular metabolic process	BP	1.98×10^{-14}	2.16×10^{-11}	4.82	115
GO:0097191	extrinsic apoptotic signaling pathway	BP	2.07×10^{-14}	2.26×10^{-11}	15.85	26
GO:0005102	signaling receptor binding	MF	2.36×10^{-14}	2.57×10^{-11}	6.54	68
GO:0001932	regulation of protein phosphorylation	BP	2.65×10^{-14}	2.89×10^{-11}	6.59	67

GO:0019220	regulation of phosphate metabolic process	BP	3.09×10^{-14}	3.36×10^{-11}	6.11	75
GO:0010605	negative regulation of macromolecule metabolic process	BP	3.32×10^{-14}	3.61×10^{-11}	5.36	93
GO:0043412	macromolecule modification	BP	3.97×10^{-14}	4.31×10^{-11}	4.69	119
GO:0030154	cell differentiation	BP	4.17×10^{-14}	4.52×10^{-11}	6.02	76
GO:0016310	phosphorylation	BP	4.80×10^{-14}	5.20×10^{-11}	5.73	82
GO:0042802	identical protein binding	MF	8.09×10^{-14}	8.76×10^{-11}	5.35	91
GO:0009892	negative regulation of metabolic process	BP	8.30×10^{-14}	8.98×10^{-11}	5.13	98
GO:0031323	regulation of cellular metabolic process	BP	9.57×10^{-14}	1.03×10^{-10}	4.11	153
GO:0010604	positive regulation of macromolecule metabolic process	BP	9.70×10^{-14}	1.05×10^{-10}	4.73	114
GO:0019222	regulation of metabolic process	BP	1.04×10^{-13}	1.12×10^{-10}	4.04	159
GO:0009893	positive regulation of metabolic process	BP	1.40×10^{-13}	1.51×10^{-10}	4.63	118
GO:0060255	regulation of macromolecule metabolic process	BP	1.73×10^{-13}	1.86×10^{-10}	4.11	151
GO:0009057	macromolecule catabolic process	BP	2.16×10^{-13}	2.33×10^{-10}	7.22	55
GO:0051171	regulation of nitrogen compound metabolic process	BP	2.41×10^{-13}	2.59×10^{-10}	4.15	146
GO:0032879	regulation of localization	BP	2.51×10^{-13}	2.70×10^{-10}	5.25	91
GO:0001934	positive regulation of protein phosphorylation	BP	2.97×10^{-13}	3.18×10^{-10}	7.46	52
GO:1901564	organonitrogen compound metabolic process	BP	3.56×10^{-13}	3.81×10^{-10}	4.05	153
GO:0051172	negative regulation of nitrogen compound metabolic	BP	3.66×10^{-13}	3.92×10^{-10}	5.41	85
GO:0006508	proteolysis	BP	4.04×10^{-13}	4.32×10^{-10}	6.41	64
GO:0050793	regulation of developmental process	BP	4.91×10^{-13}	5.25×10^{-10}	5.98	71
GO:0080090	regulation of primary metabolic process	BP	5.63×10^{-13}	6.02×10^{-10}	4.10	147
GO:0051248	negative regulation of protein metabolic process	BP	8.55×10^{-13}	9.12×10^{-10}	7.16	53
GO:0045937	positive regulation of phosphate metabolic process	BP	9.36×10^{-13}	9.98×10^{-10}	6.96	55
GO:0042176	regulation of protein catabolic process	BP	9.43×10^{-13}	1.00×10^{-9}	11.23	31
GO:0019904	protein domain specific binding	MF	1.33×10^{-12}	1.42×10^{-9}	7.99	45
GO:2001235	positive regulation of apoptotic signaling pathway	BP	1.60×10^{-12}	1.70×10^{-9}	17.07	21
GO:0006793	phosphorus metabolic process	BP	1.69×10^{-12}	1.80×10^{-9}	4.97	95
GO:0048585	negative regulation of response to stimulus	BP	2.34×10^{-12}	2.49×10^{-9}	6.17	64
GO:0002682	regulation of immune system process	BP	2.42×10^{-12}	2.57×10^{-9}	6.80	55
GO:0042127	regulation of cell proliferation	BP	2.44×10^{-12}	2.58×10^{-9}	6.42	60
GO:0031324	negative regulation of cellular metabolic process	BP	2.53×10^{-12}	2.68×10^{-9}	5.13	88
GO:0033554	cellular response to stress	BP	3.01×10^{-12}	3.19×10^{-9}	5.67	73
GO:1903362	regulation of cellular protein catabolic process	BP	3.46×10^{-12}	3.65×10^{-9}	13.37	25
GO:0033993	response to lipid	BP	4.87×10^{-12}	5.14×10^{-9}	9.00	37
GO:0051239	regulation of multicellular organismal process	BP	6.19×10^{-12}	6.52×10^{-9}	5.31	80
GO:0044093	positive regulation of molecular function	BP	8.93×10^{-12}	9.40×10^{-9}	5.87	66
GO:0044260	cellular macromolecule metabolic process	BP	9.48×10^{-12}	9.97×10^{-9}	3.71	173
GO:0030163	protein catabolic process	BP	9.75×10^{-12}	1.02×10^{-8}	7.80	43
GO:1903320	regulation of protein modification by small protein conjugation or removal	BP	9.96×10^{-12}	1.05×10^{-8}	12.76	25
GO:1902533	positive regulation of intracellular signal transduction	BP	1.57×10^{-11}	1.64×10^{-8}	6.91	50
GO:0080135	regulation of cellular response to stress	BP	2.24×10^{-11}	2.35×10^{-8}	7.88	41
GO:0002684	positive regulation of immune system process	BP	2.34×10^{-11}	2.45×10^{-8}	7.47	44
GO:0023057	negative regulation of signaling	BP	2.53×10^{-11}	2.65×10^{-8}	6.40	55
GO:0051130	positive regulation of cellular component organization	BP	3.49×10^{-11}	3.64×10^{-8}	6.67	51
GO:0010648	negative regulation of cell communication	BP	4.86×10^{-11}	5.08×10^{-8}	6.37	54
GO:0043170	macromolecule metabolic process	BP	4.92×10^{-11}	5.13×10^{-8}	3.54	186
GO:2001236	regulation of extrinsic apoptotic signaling pathway	BP	5.46×10^{-11}	5.69×10^{-8}	16.10	19
GO:0052547	regulation of peptidase activity	BP	7.04×10^{-11}	7.33×10^{-8}	10.45	28
GO:0044257	cellular protein catabolic process	BP	7.88×10^{-11}	8.19×10^{-8}	8.19	37
GO:0050790	regulation of catalytic activity	BP	1.04×10^{-10}	1.08×10^{-7}	5.38	70
GO:0044087	regulation of cellular component biogenesis	BP	1.18×10^{-10}	1.22×10^{-7}	6.90	46
GO:0016477	cell migration	BP	1.21×10^{-10}	1.26×10^{-7}	6.15	55

GO:0007275	multicellular organism development	BP	1.31×10^{-10}	1.35×10^{-7}	4.91	83
GO:0031329	regulation of cellular catabolic process	BP	1.43×10^{-10}	1.48×10^{-7}	7.56	40
GO:0044265	cellular macromolecule catabolic process	BP	1.45×10^{-10}	1.50×10^{-7}	6.96	45
GO:0009968	negative regulation of signal transduction	BP	1.49×10^{-10}	1.53×10^{-7}	6.33	52
GO:0032269	negative regulation of cellular protein metabolic process	BP	1.54×10^{-10}	1.59×10^{-7}	6.74	47
GO:0009894	regulation of catabolic process	BP	1.60×10^{-10}	1.65×10^{-7}	7.15	43
GO:1901700	response to oxygen-containing compound	BP	1.96×10^{-10}	2.02×10^{-7}	6.70	47
GO:0018193	peptidyl-amino acid modification	BP	2.82×10^{-10}	2.91×10^{-7}	6.29	51
GO:0051336	regulation of hydrolase activity	BP	3.27×10^{-10}	3.36×10^{-7}	6.69	46
GO:1901565	organonitrogen compound catabolic process	BP	3.45×10^{-10}	3.54×10^{-7}	6.58	47
GO:0005126	cytokine receptor binding	MF	3.48×10^{-10}	3.57×10^{-7}	12.96	21
GO:0045862	positive regulation of proteolysis	BP	3.87×10^{-10}	3.96×10^{-7}	10.04	27
GO:0016032	viral process	BP	4.10×10^{-10}	4.20×10^{-7}	9.69	28
GO:1901701	cellular response to oxygen-containing compound	BP	4.57×10^{-10}	4.68×10^{-7}	7.27	40
GO:0052548	regulation of endopeptidase activity	BP	4.95×10^{-10}	5.06×10^{-7}	10.28	26
GO:0071396	cellular response to lipid	BP	5.18×10^{-10}	5.29×10^{-7}	9.04	30
GO:0019900	kinase binding	MF	5.49×10^{-10}	5.60×10^{-7}	6.99	42
GO:0097193	intrinsic apoptotic signaling pathway	BP	5.79×10^{-10}	5.90×10^{-7}	11.01	24
GO:0043254	regulation of protein complex assembly	BP	6.60×10^{-10}	6.72×10^{-7}	9.21	29
GO:0014070	response to organic cyclic compound	BP	6.81×10^{-10}	6.93×10^{-7}	8.27	33
GO:0030334	regulation of cell migration	BP	7.14×10^{-10}	7.26×10^{-7}	6.72	44
GO:0098609	cell-cell adhesion	BP	7.26×10^{-10}	7.37×10^{-7}	8.25	33
GO:0044248	cellular catabolic process	BP	8.01×10^{-10}	8.12×10^{-7}	5.29	66
GO:0008284	positive regulation of cell proliferation	BP	8.18×10^{-10}	8.29×10^{-7}	7.13	40
GO:0044459	plasma membrane part	CC	8.47×10^{-10}	8.57×10^{-7}	5.46	62
GO:0071407	cellular response to organic cyclic compound	BP	8.56×10^{-10}	8.65×10^{-7}	8.85	30
GO:0031396	regulation of protein ubiquitination	BP	8.59×10^{-10}	8.68×10^{-7}	12.35	21
GO:0071495	cellular response to endogenous stimulus	BP	9.51×10^{-10}	9.58×10^{-7}	6.47	46
GO:0006952	defense response	BP	9.50×10^{-10}	9.59×10^{-7}	6.66	44
GO:0034097	response to cytokine	BP	1.06×10^{-9}	1.07×10^{-6}	7.94	34
GO:0051049	regulation of transport	BP	1.25×10^{-9}	1.26×10^{-6}	5.65	57
GO:1903050	regulation of proteolysis involved in cellular protein catabolic process	BP	1.26×10^{-9}	1.27×10^{-6}	12.74	20
GO:0002020	protease binding	MF	1.33×10^{-9}	1.33×10^{-6}	14.29	18
GO:0000165	MAPK cascade	BP	1.36×10^{-9}	1.36×10^{-6}	7.13	39
GO:2000116	regulation of cysteine-type endopeptidase activity	BP	1.62×10^{-9}	1.63×10^{-6}	10.90	23
GO:0043933	protein-containing complex subunit organization	BP	1.64×10^{-9}	1.64×10^{-6}	5.24	65
GO:0045595	regulation of cell differentiation	BP	2.51×10^{-9}	2.51×10^{-6}	6.28	46
GO:0030155	regulation of cell adhesion	BP	2.91×10^{-9}	2.91×10^{-6}	7.64	34
GO:0044403	symbiont process	BP	2.92×10^{-9}	2.91×10^{-6}	8.40	30
GO:0044092	negative regulation of molecular function	BP	3.13×10^{-9}	3.12×10^{-6}	6.15	47
GO:0006810	transport	BP	3.14×10^{-9}	3.13×10^{-6}	4.36	94
GO:2001238	positive regulation of extrinsic apoptotic signaling	BP	4.03×10^{-9}	4.01×10^{-6}	25.58	11
GO:2000377	regulation of reactive oxygen species metabolic process	BP	4.29×10^{-9}	4.26×10^{-6}	16.48	15
GO:0009653	anatomical structure morphogenesis	BP	4.42×10^{-9}	4.39×10^{-6}	5.63	54
GO:0010498	proteasomal protein catabolic process	BP	5.87×10^{-9}	5.82×10^{-6}	9.47	25
GO:2001242	regulation of intrinsic apoptotic signaling pathway	BP	5.88×10^{-9}	5.83×10^{-6}	13.04	18
GO:0070997	neuron death	BP	6.42×10^{-9}	6.36×10^{-6}	13.82	17
GO:0040012	regulation of locomotion	BP	6.91×10^{-9}	6.83×10^{-6}	6.16	45
GO:0043161	proteasome-mediated ubiquitin-dependent protein catabolic process	BP	7.12×10^{-9}	7.03×10^{-6}	10.53	22
GO:1902532	negative regulation of intracellular signal transduction	BP	7.86×10^{-9}	7.76×10^{-6}	7.67	32
GO:0065003	protein-containing complex assembly	BP	7.92×10^{-9}	7.81×10^{-6}	5.28	59
GO:0006898	receptor-mediated endocytosis	BP	9.32×10^{-9}	9.18×10^{-6}	13.49	17

GO:1901575	organic substance catabolic process	BP	9.50×10^{-9}	9.35×10^{-6}	5.30	58
GO:2000026	regulation of multicellular organismal development	BP	9.63×10^{-9}	9.46×10^{-6}	5.73	50
GO:0051603	proteolysis involved in cellular protein catabolic process	BP	9.62×10^{-9}	9.46×10^{-6}	7.79	31
GO:0045935	positive regulation of nucleobase-containing compound metabolic process	BP	1.06×10^{-8}	1.04×10^{-5}	4.86	69
GO:0006511	ubiquitin-dependent protein catabolic process	BP	1.22×10^{-8}	1.20×10^{-5}	8.57	27
GO:0006954	inflammatory response	BP	1.39×10^{-8}	1.36×10^{-5}	11.11	20
GO:2001234	negative regulation of apoptotic signaling pathway	BP	1.46×10^{-8}	1.43×10^{-5}	11.66	19
GO:0043408	regulation of MAPK cascade	BP	1.53×10^{-8}	1.50×10^{-5}	7.01	35
GO:0022607	cellular component assembly	BP	1.63×10^{-8}	1.59×10^{-5}	4.53	79
GO:0010629	negative regulation of gene expression	BP	1.67×10^{-8}	1.63×10^{-5}	5.13	60
GO:0048660	regulation of smooth muscle cell proliferation	BP	1.68×10^{-8}	1.63×10^{-5}	16.28	14
GO:0006897	endocytosis	BP	1.69×10^{-8}	1.64×10^{-5}	9.30	24
GO:0043900	regulation of multi-organism process	BP	1.77×10^{-8}	1.72×10^{-5}	8.19	28
GO:0051094	positive regulation of developmental process	BP	1.83×10^{-8}	1.77×10^{-5}	6.30	41
GO:0018105	peptidyl-serine phosphorylation	BP	1.83×10^{-8}	1.78×10^{-5}	10.00	22
GO:0071944	cell periphery	CC	1.88×10^{-8}	1.82×10^{-5}	4.05	104
GO:0098657	import into cell	BP	1.97×10^{-8}	1.90×10^{-5}	8.64	26
GO:0005886	plasma membrane	CC	1.99×10^{-8}	1.92×10^{-5}	4.07	102
GO:0043281	regulation of cysteine-type endopeptidase activity involved in apoptotic process	BP	2.22×10^{-8}	2.14×10^{-5}	10.81	20
GO:0002683	negative regulation of immune system process	BP	2.45×10^{-8}	2.37×10^{-5}	9.13	24
GO:0009891	positive regulation of biosynthetic process	BP	3.02×10^{-8}	2.92×10^{-5}	4.77	68
GO:0051240	positive regulation of multicellular organismal process	BP	3.31×10^{-8}	3.18×10^{-5}	5.52	50
GO:0032446	protein modification by small protein conjugation	BP	3.42×10^{-8}	3.29×10^{-5}	6.92	34
GO:0043085	positive regulation of catalytic activity	BP	3.45×10^{-8}	3.32×10^{-5}	5.70	47
GO:0043901	negative regulation of multi-organism process	BP	3.74×10^{-8}	3.59×10^{-5}	11.61	18
GO:0045732	positive regulation of protein catabolic process	BP	3.74×10^{-8}	3.59×10^{-5}	11.61	18
GO:1903322	positive regulation of protein modification by small protein conjugation or removal	BP	3.85×10^{-8}	3.70×10^{-5}	13.11	16
GO:0018108	peptidyl-tyrosine phosphorylation	BP	3.86×10^{-8}	3.70×10^{-5}	9.24	23
GO:0035821	modification of morphology or physiology of other	BP	4.14×10^{-8}	3.96×10^{-5}	11.54	18
GO:2000058	regulation of ubiquitin-dependent protein catabolic	BP	4.16×10^{-8}	3.97×10^{-5}	14.02	15
GO:0035966	response to topologically incorrect protein	BP	4.17×10^{-8}	3.99×10^{-5}	18.46	12
GO:0009725	response to hormone	BP	4.20×10^{-8}	4.00×10^{-5}	8.08	27
GO:0043086	negative regulation of catalytic activity	BP	5.07×10^{-8}	4.83×10^{-5}	6.81	34
GO:0043491	protein kinase B signaling	BP	7.04×10^{-8}	6.70×10^{-5}	11.81	17
GO:0009986	cell surface	CC	8.33×10^{-8}	7.92×10^{-5}	6.80	33
GO:0032101	regulation of response to external stimulus	BP	8.49×10^{-8}	8.07×10^{-5}	7.25	30
GO:1901698	response to nitrogen compound	BP	8.74×10^{-8}	8.30×10^{-5}	6.79	33
GO:0019219	regulation of nucleobase-containing compound metabolic process	BP	9.85×10^{-8}	9.34×10^{-5}	4.02	97
GO:1903364	positive regulation of cellular protein catabolic process	BP	1.06×10^{-7}	1.00×10^{-4}	14.14	14
GO:0019955	cytokine binding	MF	1.18×10^{-7}	1.11×10^{-4}	15.29	13
GO:0008285	negative regulation of cell proliferation	BP	1.19×10^{-7}	1.12×10^{-4}	7.30	29
GO:0016567	protein ubiquitination	BP	1.24×10^{-7}	1.17×10^{-4}	6.97	31
GO:1901214	regulation of neuron death	BP	1.36×10^{-7}	1.29×10^{-4}	13.86	14
GO:0051050	positive regulation of transport	BP	1.38×10^{-7}	1.30×10^{-4}	6.21	37
GO:2000379	positive regulation of reactive oxygen species metabolic	BP	1.39×10^{-7}	1.31×10^{-4}	18.64	11
GO:0034976	response to endoplasmic reticulum stress	BP	1.42×10^{-7}	1.34×10^{-4}	10.65	18
GO:0010468	regulation of gene expression	BP	1.52×10^{-7}	1.42×10^{-4}	3.93	101
GO:0008104	protein localization	BP	1.62×10^{-7}	1.52×10^{-4}	4.68	64
GO:0016192	vesicle-mediated transport	BP	1.77×10^{-7}	1.65×10^{-4}	5.96	39

GO:0071345	cellular response to cytokine stimulus	BP	1.94×10^{-7}	1.82×10^{-4}	7.50	27
GO:1904705	regulation of vascular smooth muscle cell proliferation	BP	2.12×10^{-7}	1.98×10^{-4}	20.41	10
GO:0019901	protein kinase binding	MF	2.14×10^{-7}	1.99×10^{-4}	6.42	34
GO:0010952	positive regulation of peptidase activity	BP	2.28×10^{-7}	2.13×10^{-4}	10.90	17
GO:0030336	negative regulation of cell migration	BP	2.31×10^{-7}	2.15×10^{-4}	9.01	21
GO:0051707	response to other organism	BP	2.34×10^{-7}	2.17×10^{-4}	6.51	33
GO:0009896	positive regulation of catabolic process	BP	2.44×10^{-7}	2.27×10^{-4}	7.84	25
GO:0050730	regulation of peptidyl-tyrosine phosphorylation	BP	2.63×10^{-7}	2.44×10^{-4}	9.74	19
GO:0050778	positive regulation of immune response	BP	3.00×10^{-7}	2.79×10^{-4}	7.54	26
GO:0031400	negative regulation of protein modification process	BP	3.03×10^{-7}	2.81×10^{-4}	6.83	30
GO:0008625	extrinsic apoptotic signaling pathway via death domain	BP	3.31×10^{-7}	3.06×10^{-4}	17.19	11
GO:0048731	system development	BP	3.38×10^{-7}	3.13×10^{-4}	4.42	70
GO:0040013	negative regulation of locomotion	BP	3.81×10^{-7}	3.52×10^{-4}	8.43	22
GO:0045861	negative regulation of proteolysis	BP	3.90×10^{-7}	3.60×10^{-4}	9.50	19
GO:1901216	positive regulation of neuron death	BP	3.94×10^{-7}	3.63×10^{-4}	26.67	8
GO:0048545	response to steroid hormone	BP	3.94×10^{-7}	3.63×10^{-4}	10.49	17
GO:0010557	positive regulation of macromolecule biosynthetic	BP	4.31×10^{-7}	3.97×10^{-4}	4.59	63
GO:0045597	positive regulation of cell differentiation	BP	4.73×10^{-7}	4.35×10^{-4}	6.84	29
GO:0070647	protein modification by small protein conjugation or	BP	4.89×10^{-7}	4.49×10^{-4}	5.99	36
GO:0043410	positive regulation of MAPK cascade	BP	4.93×10^{-7}	4.52×10^{-4}	7.34	26
GO:0042177	negative regulation of protein catabolic process	BP	5.62×10^{-7}	5.15×10^{-4}	12.39	14
GO:0019838	growth factor binding	MF	5.72×10^{-7}	5.23×10^{-4}	13.40	13
GO:0050776	regulation of immune response	BP	5.74×10^{-7}	5.25×10^{-4}	6.78	29
GO:1905897	regulation of response to endoplasmic reticulum stress	BP	6.26×10^{-7}	5.71×10^{-4}	16.18	11
GO:0097435	supramolecular fiber organization	BP	6.30×10^{-7}	5.75×10^{-4}	7.07	27
GO:0009889	regulation of biosynthetic process	BP	7.46×10^{-7}	6.80×10^{-4}	3.90	94
GO:0007167	enzyme linked receptor protein signaling pathway	BP	7.73×10^{-7}	7.03×10^{-4}	6.29	32
GO:0009888	tissue development	BP	8.00×10^{-7}	7.27×10^{-4}	5.70	38
GO:0033138	positive regulation of peptidyl-serine phosphorylation	BP	9.18×10^{-7}	8.33×10^{-4}	12.87	13
GO:0010628	positive regulation of gene expression	BP	9.18×10^{-7}	8.33×10^{-4}	4.43	65
GO:0051098	regulation of binding	BP	9.21×10^{-7}	8.35×10^{-4}	7.74	23
GO:0031331	positive regulation of cellular catabolic process	BP	9.27×10^{-7}	8.39×10^{-4}	8.00	22
GO:0036462	TRAIL-activated apoptotic signaling pathway	BP	9.31×10^{-7}	8.41×10^{-4}	55.56	5
GO:0051252	regulation of RNA metabolic process	BP	9.41×10^{-7}	8.50×10^{-4}	3.98	87
GO:0007249	I-kappaB kinase/NF-kappaB signaling	BP	9.61×10^{-7}	8.66×10^{-4}	9.38	18
GO:0050731	positive regulation of peptidyl-tyrosine phosphorylation	BP	1.01×10^{-6}	9.11×10^{-4}	11.03	15
GO:0007267	cell-cell signaling	BP	1.02×10^{-6}	9.18×10^{-4}	6.45	30
GO:0006996	organelle organization	BP	1.04×10^{-6}	9.33×10^{-4}	3.97	87
GO:0001704	formation of primary germ layer	BP	1.04×10^{-6}	9.34×10^{-4}	20.00	9
GO:0032459	regulation of protein oligomerization	BP	1.04×10^{-6}	9.34×10^{-4}	20.00	9
GO:0009895	negative regulation of catabolic process	BP	1.12×10^{-6}	1.00×10^{-3}	8.51	20
GO:0035987	endodermal cell differentiation	BP	1.12×10^{-6}	1.01×10^{-3}	23.53	8
GO:0019058	viral life cycle	BP	1.18×10^{-6}	1.06×10^{-3}	8.84	19
GO:0070371	ERK1 and ERK2 cascade	BP	1.19×10^{-6}	1.07×10^{-3}	8.47	20
GO:1990381	ubiquitin-specific protease binding	MF	1.24×10^{-6}	1.11×10^{-3}	37.50	6
GO:0033043	regulation of organelle organization	BP	1.25×10^{-6}	1.11×10^{-3}	5.14	45
GO:0051090	regulation of DNA-binding transcription factor activity	BP	1.26×10^{-6}	1.13×10^{-3}	6.99	26
GO:0032092	positive regulation of protein binding	BP	1.29×10^{-6}	1.15×10^{-3}	13.64	12
GO:0010638	positive regulation of organelle organization	BP	1.33×10^{-6}	1.18×10^{-3}	6.37	30
GO:0010243	response to organonitrogen compound	BP	1.40×10^{-6}	1.24×10^{-3}	6.78	27
GO:0051253	negative regulation of RNA metabolic process	BP	1.45×10^{-6}	1.29×10^{-3}	5.11	45
GO:1901576	organic substance biosynthetic process	BP	1.49×10^{-6}	1.32×10^{-3}	3.62	113
GO:0031347	regulation of defense response	BP	1.52×10^{-6}	1.35×10^{-3}	7.10	25
GO:0071705	nitrogen compound transport	BP	1.64×10^{-6}	1.45×10^{-3}	4.99	47
GO:0071214	cellular response to abiotic stimulus	BP	1.67×10^{-6}	1.47×10^{-3}	8.64	19

GO:0022603	regulation of anatomical structure morphogenesis	BP	1.69×10^{-6}	1.49×10^{-3}	6.18	31
GO:0031326	regulation of cellular biosynthetic process	BP	1.71×10^{-6}	1.51×10^{-3}	3.85	92
GO:0008630	intrinsic apoptotic signaling pathway in response to DNA damage	BP	1.72×10^{-6}	1.51×10^{-3}	14.67	11
GO:0051219	phosphoprotein binding	MF	1.72×10^{-6}	1.51×10^{-3}	14.67	11
GO:0061136	regulation of proteasomal protein catabolic process	BP	1.75×10^{-6}	1.54×10^{-3}	11.29	14
GO:0060341	regulation of cellular localization	BP	1.78×10^{-6}	1.56×10^{-3}	6.05	32
GO:0010466	negative regulation of peptidase activity	BP	1.79×10^{-6}	1.57×10^{-3}	12.15	13
GO:0071702	organic substance transport	BP	1.82×10^{-6}	1.60×10^{-3}	4.72	53
GO:0031349	positive regulation of defense response	BP	1.91×10^{-6}	1.67×10^{-3}	8.56	19
GO:0043903	regulation of symbiosis. encompassing mutualism through parasitism	BP	1.91×10^{-6}	1.67×10^{-3}	8.56	19
GO:0051896	regulation of protein kinase B signaling	BP	1.93×10^{-6}	1.69×10^{-3}	11.20	14
GO:0009059	macromolecule biosynthetic process	BP	1.93×10^{-6}	1.69×10^{-3}	3.73	101
GO:0031330	negative regulation of cellular catabolic process	BP	2.15×10^{-6}	1.88×10^{-3}	8.87	18
GO:0034645	cellular macromolecule biosynthetic process	BP	2.15×10^{-6}	1.88×10^{-3}	3.74	99
GO:0045934	negative regulation of nucleobase-containing compound metabolic process	BP	2.19×10^{-6}	1.91×10^{-3}	4.85	49
GO:0010556	regulation of macromolecule biosynthetic process	BP	2.32×10^{-6}	2.02×10^{-3}	3.85	90
GO:0043393	regulation of protein binding	BP	2.36×10^{-6}	2.05×10^{-3}	9.24	17
GO:0042493	response to drug	BP	2.39×10^{-6}	2.07×10^{-3}	6.75	26
GO:0005615	extracellular space	CC	2.39×10^{-6}	2.07×10^{-3}	5.44	38
GO:0051259	protein complex oligomerization	BP	2.57×10^{-6}	2.22×10^{-3}	6.57	27
GO:0006935	chemotaxis	BP	2.74×10^{-6}	2.36×10^{-3}	7.75	21
GO:0010035	response to inorganic substance	BP	2.74×10^{-6}	2.36×10^{-3}	7.75	21
GO:0009890	negative regulation of biosynthetic process	BP	2.81×10^{-6}	2.43×10^{-3}	4.80	49
GO:0051345	positive regulation of hydrolase activity	BP	2.95×10^{-6}	2.54×10^{-3}	6.52	27
GO:2000112	regulation of cellular macromolecule biosynthetic process	BP	2.98×10^{-6}	2.56×10^{-3}	3.86	88
GO:0051254	positive regulation of RNA metabolic process	BP	3.00×10^{-6}	2.58×10^{-3}	4.50	57
GO:0051817	modification of morphology or physiology of other organism involved in symbiotic interaction	BP	3.01×10^{-6}	2.59×10^{-3}	11.61	13
GO:0007159	leukocyte cell-cell adhesion	BP	3.05×10^{-6}	2.62×10^{-3}	8.65	18
GO:0050900	leukocyte migration	BP	3.05×10^{-6}	2.62×10^{-3}	8.65	18
GO:0032870	cellular response to hormone stimulus	BP	3.14×10^{-6}	2.69×10^{-3}	7.43	22
GO:0002761	regulation of myeloid leukocyte differentiation	BP	3.27×10^{-6}	2.80×10^{-3}	15.38	10
GO:0043112	receptor metabolic process	BP	3.39×10^{-6}	2.90×10^{-3}	10.69	14
GO:0048468	cell development	BP	3.51×10^{-6}	2.99×10^{-3}	6.07	30
GO:0010467	gene expression	BP	3.66×10^{-6}	3.12×10^{-3}	3.59	109
GO:2001243	negative regulation of intrinsic apoptotic signaling	BP	3.74×10^{-6}	3.19×10^{-3}	13.58	11
GO:0009636	response to toxic substance	BP	4.13×10^{-6}	3.51×10^{-3}	9.87	15
GO:0048525	negative regulation of viral process	BP	4.59×10^{-6}	3.90×10^{-3}	12.12	12
GO:0097237	cellular response to toxic substance	BP	4.59×10^{-6}	3.90×10^{-3}	12.12	12
GO:0034612	response to tumor necrosis factor	BP	4.61×10^{-6}	3.92×10^{-3}	9.25	16
GO:0045893	positive regulation of transcription. DNA-templated	BP	4.88×10^{-6}	4.14×10^{-3}	4.53	54
GO:0071363	cellular response to growth factor stimulus	BP	5.02×10^{-6}	4.25×10^{-3}	6.48	26
GO:0031398	positive regulation of protein ubiquitination	BP	5.10×10^{-6}	4.31×10^{-3}	12.00	12
GO:0051338	regulation of transferase activity	BP	5.10×10^{-6}	4.31×10^{-3}	5.41	36
GO:0005737	cytoplasm	CC	5.20×10^{-6}	4.39×10^{-3}	3.02	205
GO:0071216	cellular response to biotic stimulus	BP	5.28×10^{-6}	4.45×10^{-3}	10.29	14
GO:0051347	positive regulation of transferase activity	BP	5.47×10^{-6}	4.61×10^{-3}	6.31	27
GO:0006919	activation of cysteine-type endopeptidase activity involved in apoptotic process	BP	5.71×10^{-6}	4.80×10^{-3}	14.49	10

GO:0051346	negative regulation of hydrolase activity	BP	5.94×10^{-6}	4.99×10^{-3}	7.63	20
GO:0051897	positive regulation of protein kinase B signaling	BP	6.04×10^{-6}	5.06×10^{-3}	12.94	11
GO:0032103	positive regulation of response to external stimulus	BP	6.20×10^{-6}	5.19×10^{-3}	9.04	16
GO:0000151	ubiquitin ligase complex	CC	6.30×10^{-6}	5.27×10^{-3}	8.22	18
GO:0019221	cytokine-mediated signaling pathway	BP	6.38×10^{-6}	5.33×10^{-3}	8.59	17
GO:0060627	regulation of vesicle-mediated transport	BP	6.79×10^{-6}	5.67×10^{-3}	7.85	19
GO:0001817	regulation of cytokine production	BP	7.18×10^{-6}	5.99×10^{-3}	6.09	28
GO:0002237	response to molecule of bacterial origin	BP	7.19×10^{-6}	5.99×10^{-3}	9.43	15
GO:0050792	regulation of viral process	BP	8.33×10^{-6}	6.93×10^{-3}	8.42	17
GO:0044089	positive regulation of cellular component biogenesis	BP	8.43×10^{-6}	7.00×10^{-3}	6.61	24
GO:0034613	cellular protein localization	BP	8.44×10^{-6}	7.01×10^{-3}	4.78	45
GO:0002520	immune system development	BP	8.56×10^{-6}	7.10×10^{-3}	6.78	23
GO:0032989	cellular component morphogenesis	BP	8.56×10^{-6}	7.10×10^{-3}	6.78	23
GO:0051241	negative regulation of multicellular organismal process	BP	8.74×10^{-6}	7.24×10^{-3}	5.60	32
GO:0045121	membrane raft	CC	8.90×10^{-6}	7.36×10^{-3}	8.37	17
GO:0034599	cellular response to oxidative stress	BP	9.03×10^{-6}	7.46×10^{-3}	9.26	15
GO:0008134	transcription factor binding	MF	9.30×10^{-6}	7.67×10^{-3}	5.78	30
GO:0002573	myeloid leukocyte differentiation	BP	9.38×10^{-6}	7.72×10^{-3}	11.32	12
GO:0031327	negative regulation of cellular biosynthetic process	BP	9.38×10^{-6}	7.73×10^{-3}	4.68	47
GO:0002764	immune response-regulating signaling pathway	BP	9.50×10^{-6}	7.81×10^{-3}	8.33	17
GO:0032102	negative regulation of response to external stimulus	BP	9.73×10^{-6}	7.99×10^{-3}	9.20	15
GO:0051099	positive regulation of binding	BP	9.73×10^{-6}	7.99×10^{-3}	9.20	15
GO:0010558	negative regulation of macromolecule biosynthetic	BP	9.93×10^{-6}	8.14×10^{-3}	4.71	46
GO:0045892	negative regulation of transcription. DNA-templated	BP	1.00×10^{-5}	8.21×10^{-3}	5.00	40
GO:0070374	positive regulation of ERK1 and ERK2 cascade	BP	1.03×10^{-5}	8.40×10^{-3}	9.72	14
GO:2001141	regulation of RNA biosynthetic process	BP	1.09×10^{-5}	8.87×10^{-3}	3.88	78
GO:0044388	small protein activating enzyme binding	MF	1.11×10^{-5}	9.03×10^{-3}	57.14	4
GO:0034641	cellular nitrogen compound metabolic process	BP	1.11×10^{-5}	9.03×10^{-3}	3.42	120
GO:0032880	regulation of protein localization	BP	1.12×10^{-5}	9.12×10^{-3}	5.30	35
GO:0048015	phosphatidylinositol-mediated signaling	BP	1.14×10^{-5}	9.26×10^{-3}	11.11	12
GO:1901699	cellular response to nitrogen compound	BP	1.19×10^{-5}	9.69×10^{-3}	6.65	23
GO:0050839	cell adhesion molecule binding	MF	1.20×10^{-5}	9.76×10^{-3}	9.59	14
GO:0044249	cellular biosynthetic process	BP	1.29×10^{-5}	1.05×10^{-2}	3.51	108
GO:0034614	cellular response to reactive oxygen species	BP	1.31×10^{-5}	1.06×10^{-2}	11.96	11
GO:0031264	death-inducing signaling complex	CC	1.34×10^{-5}	1.08×10^{-2}	35.71	5
GO:0051788	response to misfolded protein	BP	1.34×10^{-5}	1.08×10^{-2}	35.71	5
GO:0006355	regulation of transcription, DNA-templated	BP	1.35×10^{-5}	1.09×10^{-2}	3.87	77
GO:0032461	positive regulation of protein oligomerization	BP	1.36×10^{-5}	1.09×10^{-2}	26.09	6
GO:0043549	regulation of kinase activity	BP	1.39×10^{-5}	1.12×10^{-2}	5.57	31
GO:0051051	negative regulation of transport	BP	1.39×10^{-5}	1.12×10^{-2}	6.98	21
GO:0035877	death effector domain binding	MF	1.40×10^{-5}	1.12×10^{-2}	100.00	3
GO:0042886	amide transport	BP	1.42×10^{-5}	1.14×10^{-2}	4.93	40
GO:0002253	activation of immune response	BP	1.43×10^{-5}	1.15×10^{-2}	7.45	19
GO:0060389	pathway-restricted SMAD protein phosphorylation	BP	1.48×10^{-5}	1.18×10^{-2}	17.02	8
GO:0044271	cellular nitrogen compound biosynthetic process	BP	1.55×10^{-5}	1.24×10^{-2}	3.63	94
GO:0090100	positive regulation of transmembrane receptor protein serine/threonine kinase signaling pathway	BP	1.66×10^{-5}	1.32×10^{-2}	14.52	9
GO:2000117	negative regulation of cysteine-type endopeptidase	BP	1.74×10^{-5}	1.39×10^{-2}	12.82	10
GO:0031647	regulation of protein stability	BP	1.78×10^{-5}	1.42×10^{-2}	7.34	19
GO:0045637	regulation of myeloid cell differentiation	BP	1.85×10^{-5}	1.47×10^{-2}	9.85	13
GO:0030097	hemopoiesis	BP	1.87×10^{-5}	1.49×10^{-2}	6.84	21
GO:1903363	negative regulation of cellular protein catabolic process	BP	1.95×10^{-5}	1.55×10^{-2}	12.66	10
GO:1902105	regulation of leukocyte differentiation	BP	1.98×10^{-5}	1.57×10^{-2}	10.53	12

GO:0045859	regulation of protein kinase activity	BP	2.00×10^{-5}	1.58×10^{-2}	5.65	29
GO:0090304	nucleic acid metabolic process	BP	2.02×10^{-5}	1.59×10^{-2}	3.52	103
GO:0048661	positive regulation of smooth muscle cell proliferation	BP	2.03×10^{-5}	1.61×10^{-2}	16.33	8
GO:0043269	regulation of ion transport	BP	2.06×10^{-5}	1.63×10^{-2}	6.80	21
GO:0010633	negative regulation of epithelial cell migration	BP	2.18×10^{-5}	1.72×10^{-2}	12.50	10
GO:0048646	anatomical structure formation involved in morphogenesis	BP	2.19×10^{-5}	1.72×10^{-2}	5.96	26
GO:1903827	regulation of cellular protein localization	BP	2.25×10^{-5}	1.77×10^{-2}	6.39	23
GO:0006914	autophagy	BP	2.27×10^{-5}	1.78×10^{-2}	6.97	20
GO:0030522	intracellular receptor signaling pathway	BP	2.28×10^{-5}	1.78×10^{-2}	8.57	15
GO:0006139	nucleobase-containing compound metabolic process	BP	2.34×10^{-5}	1.83×10^{-2}	3.45	109
GO:0035690	cellular response to drug	BP	2.39×10^{-5}	1.87×10^{-2}	8.12	16
GO:0045807	positive regulation of endocytosis	BP	2.44×10^{-5}	1.90×10^{-2}	12.35	10
GO:1903052	positive regulation of proteolysis involved in cellular protein catabolic process	BP	2.44×10^{-5}	1.90×10^{-2}	12.35	10
GO:0030099	myeloid cell differentiation	BP	2.54×10^{-5}	1.98×10^{-2}	8.08	16
GO:0043280	positive regulation of cysteine-type endopeptidase activity involved in apoptotic process	BP	2.58×10^{-5}	2.00×10^{-2}	10.26	12
GO:0051702	interaction with symbiont	BP	2.64×10^{-5}	2.05×10^{-2}	11.11	11
GO:0048729	tissue morphogenesis	BP	2.74×10^{-5}	2.13×10^{-2}	8.92	14
GO:0048598	embryonic morphogenesis	BP	2.81×10^{-5}	2.18×10^{-2}	10.17	12
GO:0045944	positive regulation of transcription by RNA polymerase II	BP	2.93×10^{-5}	2.27×10^{-2}	4.72	41
GO:0031334	positive regulation of protein complex assembly	BP	2.97×10^{-5}	2.30×10^{-2}	8.38	15
GO:0033627	cell adhesion mediated by integrin	BP	3.19×10^{-5}	2.46×10^{-2}	15.38	8
GO:0022407	regulation of cell-cell adhesion	BP	3.23×10^{-5}	2.49×10^{-2}	7.29	18
GO:0051272	positive regulation of cellular component movement	BP	3.39×10^{-5}	2.61×10^{-2}	5.94	25
GO:0015833	peptide transport	BP	3.46×10^{-5}	2.66×10^{-2}	4.84	38
GO:0071383	cellular response to steroid hormone stimulus	BP	3.46×10^{-5}	2.66×10^{-2}	9.29	13
GO:0045321	leukocyte activation	BP	3.49×10^{-5}	2.68×10^{-2}	6.38	22
GO:0002768	immune response-regulating cell surface receptor signaling pathway	BP	3.50×10^{-5}	2.68×10^{-2}	10.78	11
GO:0001540	amyloid-beta binding	MF	3.57×10^{-5}	2.73×10^{-2}	17.95	7
GO:0097718	disordered domain specific binding	MF	3.57×10^{-5}	2.73×10^{-2}	17.95	7
GO:1902930	regulation of alcohol biosynthetic process	BP	3.67×10^{-5}	2.80×10^{-2}	22.22	6
GO:0006979	response to oxidative stress	BP	3.79×10^{-5}	2.89×10^{-2}	7.49	17
GO:0048156	tau protein binding	MF	3.83×10^{-5}	2.92×10^{-2}	44.44	4
GO:0045087	innate immune response	BP	3.92×10^{-5}	2.98×10^{-2}	6.17	23
GO:0017038	protein import	BP	3.92×10^{-5}	2.98×10^{-2}	9.84	12
GO:0050865	regulation of cell activation	BP	4.08×10^{-5}	3.10×10^{-2}	6.91	19
GO:0070555	response to interleukin-1	BP	4.13×10^{-5}	3.13×10^{-2}	11.63	10
GO:0007169	transmembrane receptor protein tyrosine kinase signaling pathway	BP	4.15×10^{-5}	3.14×10^{-2}	6.48	21
GO:1903829	positive regulation of cellular protein localization	BP	4.37×10^{-5}	3.31×10^{-2}	7.73	16
GO:1901362	organic cyclic compound biosynthetic process	BP	4.40×10^{-5}	3.32×10^{-2}	3.63	86
GO:0051260	protein homooligomerization	BP	4.44×10^{-5}	3.34×10^{-2}	7.11	18
GO:0009617	response to bacterium	BP	4.47×10^{-5}	3.36×10^{-2}	6.64	20
GO:0051091	positive regulation of DNA-binding transcription factor	BP	4.48×10^{-5}	3.37×10^{-2}	7.39	17
GO:0003705	transcription factor activity. RNA polymerase II distal enhancer sequence-specific binding	MF	4.58×10^{-5}	3.44×10^{-2}	21.43	6
GO:0043123	positive regulation of I-kappaB kinase/NF-kappaB	BP	4.60×10^{-5}	3.45×10^{-2}	9.68	12
GO:0030100	regulation of endocytosis	BP	4.66×10^{-5}	3.49×10^{-2}	9.03	13
GO:0099513	polymeric cytoskeletal fiber	CC	4.73×10^{-5}	3.54×10^{-2}	6.83	19
GO:0007010	cytoskeleton organization	BP	4.88×10^{-5}	3.64×10^{-2}	4.82	37
GO:0016070	RNA metabolic process	BP	4.89×10^{-5}	3.65×10^{-2}	3.56	91

GO:1902903	regulation of supramolecular fiber organization	BP	4.99×10^{-5}	3.72×10^{-2}	7.33	17
GO:0051402	neuron apoptotic process	BP	5.03×10^{-5}	3.75×10^{-2}	12.68	9
GO:2001237	negative regulation of extrinsic apoptotic signaling	BP	5.03×10^{-5}	3.75×10^{-2}	12.68	9
GO:0033674	positive regulation of kinase activity	BP	5.14×10^{-5}	3.82×10^{-2}	6.21	22
GO:0070513	death domain binding	MF	5.29×10^{-5}	3.92×10^{-2}	27.78	5
GO:0051701	interaction with host	BP	5.39×10^{-5}	4.00×10^{-2}	9.52	12
GO:0030335	positive regulation of cell migration	BP	5.44×10^{-5}	4.02×10^{-2}	5.90	24
GO:0071248	cellular response to metal ion	BP	5.46×10^{-5}	4.04×10^{-2}	10.28	11
GO:0071316	cellular response to nicotine	BP	5.49×10^{-5}	4.05×10^{-2}	75.00	3
GO:0097199	cysteine-type endopeptidase activity involved in apoptotic signaling pathway	MF	5.49×10^{-5}	4.05×10^{-2}	75.00	3
GO:1990452	Parkin-FBXW7-Cul1 ubiquitin ligase complex	CC	5.49×10^{-5}	4.05×10^{-2}	75.00	3
GO:2000152	regulation of ubiquitin-specific protease activity	BP	5.49×10^{-5}	4.05×10^{-2}	75.00	3
GO:0006366	transcription by RNA polymerase II	BP	5.57×10^{-5}	4.10×10^{-2}	4.10	56
GO:0018130	heterocycle biosynthetic process	BP	5.59×10^{-5}	4.11×10^{-2}	3.63	84
GO:0005874	microtubule	CC	5.59×10^{-5}	4.11×10^{-2}	7.94	15
GO:0019956	chemokine binding	MF	5.65×10^{-5}	4.15×10^{-2}	20.69	6
GO:1903706	regulation of hemopoiesis	BP	5.94×10^{-5}	4.35×10^{-2}	7.89	15
GO:0030518	intracellular steroid hormone receptor signaling pathway	BP	5.95×10^{-5}	4.36×10^{-2}	10.19	11
GO:0007569	cell aging	BP	6.13×10^{-5}	4.48×10^{-2}	11.11	10
GO:0042995	cell projection	CC	6.27×10^{-5}	4.57×10^{-2}	5.00	33
GO:0048025	negative regulation of mRNA splicing, via spliceosome	BP	6.27×10^{-5}	4.58×10^{-2}	40.00	4
GO:0009755	hormone-mediated signaling pathway	BP	6.29×10^{-5}	4.58×10^{-2}	9.38	12
GO:0032182	ubiquitin-like protein binding	MF	6.30×10^{-5}	4.58×10^{-2}	14.04	8
GO:0045184	establishment of protein localization	BP	6.87×10^{-5}	4.99×10^{-2}	4.59	40

MF (molecular function), BP (biological process), CC (cellular component)

Supplementary Table S11. GO analysis of the extended interactome of integrated colon markers linked to the long-term anti-TNF response in CD patients.

GO ID	GO term	ontology source	p-value	adjusted p-value	% assoc. genes	nr. assoc. genes
GO:0000151	ubiquitin ligase complex	CC	1.65×10^{-11}	8.27×10^{-11}	5.02	11
GO:0031461	cullin-RING ubiquitin ligase complex	CC	3.56×10^{-9}	1.42×10^{-8}	5.93	8
GO:0009416	response to light stimulus	BP	8.62×10^{-5}	2.59×10^{-4}	3.13	5
GO:0035967	cellular response to topologically incorrect protein	BP	5.34×10^{-4}	1.07×10^{-3}	5.36	3
GO:0034644	cellular response to UV	BP	1.74×10^{-3}	1.74×10^{-3}	3.57	3
GO:0050709	negative regulation of protein secretion	BP	1.74×10^{-3}	1.74×10^{-3}	3.57	3

BP (biological process), CC (cellular component)

Supplementary Table S12. GO analysis of the extended interactome of integrated blood markers linked to the short-term anti-TNF response in CD patients.

GO ID	GO term	ontology source	p-value	adjusted p-value	% assoc. genes	nr. assoc. genes
GO:0035556	intracellular signal transduction	BP	2.50×10^{-24}	1.31×10^{-21}	4.41	70
GO:0007165	signal transduction	BP	6.14×10^{-24}	3.23×10^{-21}	3.35	90
GO:0044267	cellular protein metabolic process	BP	1.31×10^{-21}	6.87×10^{-19}	3.05	92
GO:0070887	cellular response to chemical stimulus	BP	1.93×10^{-21}	1.01×10^{-18}	4.05	68
GO:1902531	regulation of intracellular signal transduction	BP	2.09×10^{-21}	1.09×10^{-18}	4.87	57
GO:0010033	response to organic substance	BP	1.70×10^{-20}	8.85×10^{-18}	4.13	64
GO:0006464	cellular protein modification process	BP	1.71×10^{-20}	8.88×10^{-18}	3.30	81
GO:0048583	regulation of response to stimulus	BP	5.40×10^{-20}	2.80×10^{-17}	3.35	78
GO:0010646	regulation of cell communication	BP	5.79×10^{-20}	3.00×10^{-17}	3.70	70
GO:0023051	regulation of signaling	BP	1.05×10^{-19}	5.42×10^{-17}	3.66	70
GO:0071310	cellular response to organic substance	BP	1.35×10^{-19}	6.94×10^{-17}	4.46	57
GO:0048584	positive regulation of response to stimulus	BP	2.61×10^{-19}	1.35×10^{-16}	4.25	59
GO:0019899	enzyme binding	MF	5.09×10^{-19}	2.62×10^{-16}	3.76	66
GO:0009967	positive regulation of signal transduction	BP	6.63×10^{-19}	3.40×10^{-16}	4.95	50
GO:0031399	regulation of protein modification process	BP	1.11×10^{-18}	5.70×10^{-16}	4.34	56
GO:0051246	regulation of protein metabolic process	BP	2.94×10^{-18}	1.50×10^{-15}	3.59	67
GO:0044389	ubiquitin-like protein ligase binding	MF	5.89×10^{-18}	3.01×10^{-15}	9.18	29
GO:0032268	regulation of cellular protein metabolic process	BP	9.77×10^{-18}	4.97×10^{-15}	3.66	64
GO:1902533	positive regulation of intracellular signal transduction	BP	1.76×10^{-17}	8.93×10^{-15}	5.66	41
GO:0007166	cell surface receptor signaling pathway	BP	5.52×10^{-16}	2.80×10^{-13}	4.05	52
GO:0007249	I-kappaB kinase/NF-kappaB signaling	BP	7.57×10^{-16}	3.83×10^{-13}	11.46	22
GO:0044093	positive regulation of molecular function	BP	1.40×10^{-15}	7.06×10^{-13}	4.27	48
GO:0097190	apoptotic signaling pathway	BP	2.37×10^{-15}	1.20×10^{-12}	7.03	30
GO:0051247	positive regulation of protein metabolic process	BP	6.57×10^{-15}	3.31×10^{-12}	4.03	49
GO:0031401	positive regulation of protein modification process	BP	2.15×10^{-14}	1.08×10^{-11}	4.61	41
GO:0051091	positive regulation of DNA-binding transcription factor activity	BP	3.41×10^{-14}	1.71×10^{-11}	9.57	22
GO:0051252	regulation of RNA metabolic process	BP	3.93×10^{-14}	1.96×10^{-11}	3.02	66
GO:0097191	extrinsic apoptotic signaling pathway	BP	6.49×10^{-14}	3.24×10^{-11}	11.59	19
GO:0006468	protein phosphorylation	BP	7.71×10^{-14}	3.84×10^{-11}	3.78	49
GO:2001141	regulation of RNA biosynthetic process	BP	1.38×10^{-13}	6.83×10^{-11}	3.08	62
GO:0006915	apoptotic process	BP	1.37×10^{-13}	6.83×10^{-11}	4.01	45
GO:0016310	phosphorylation	BP	1.94×10^{-13}	9.61×10^{-11}	3.57	51
GO:0051090	regulation of DNA-binding transcription factor activity	BP	2.38×10^{-13}	1.17×10^{-10}	6.99	26
GO:0006355	regulation of transcription, DNA-templated	BP	3.04×10^{-13}	1.50×10^{-10}	3.07	61
GO:0042325	regulation of phosphorylation	BP	7.84×10^{-13}	3.86×10^{-10}	3.97	43
GO:1903320	regulation of protein modification by small protein conjugation or removal	BP	1.67×10^{-12}	8.19×10^{-10}	9.69	19
GO:0030163	protein catabolic process	BP	1.89×10^{-12}	9.28×10^{-10}	5.44	30
GO:0019220	regulation of phosphate metabolic process	BP	3.15×10^{-12}	1.54×10^{-9}	3.66	45
GO:0019900	kinase binding	MF	3.15×10^{-12}	1.54×10^{-9}	5.16	31
GO:0001934	positive regulation of protein phosphorylation	BP	5.84×10^{-12}	2.84×10^{-9}	4.73	33
GO:0034097	response to cytokine	BP	5.90×10^{-12}	2.87×10^{-9}	6.07	26
GO:1901565	organonitrogen compound catabolic process	BP	1.12×10^{-11}	5.42×10^{-9}	4.62	33

GO:0042176	regulation of protein catabolic process	BP	1.16×10^{-11}	5.60×10^{-9}	7.61	21
GO:0009057	macromolecule catabolic process	BP	1.36×10^{-11}	6.56×10^{-9}	4.46	34
GO:0031396	regulation of protein ubiquitination	BP	1.60×10^{-11}	7.69×10^{-9}	10.00	17
GO:0019904	protein domain specific binding	MF	1.80×10^{-11}	8.67×10^{-9}	5.15	29
GO:0030162	regulation of proteolysis	BP	1.93×10^{-11}	9.25×10^{-9}	5.53	27
GO:0032446	protein modification by small protein conjugation	BP	2.22×10^{-11}	1.06×10^{-8}	5.50	27
GO:0071495	cellular response to endogenous stimulus	BP	4.81×10^{-11}	2.30×10^{-8}	4.50	32
GO:0033554	cellular response to stress	BP	6.06×10^{-11}	2.89×10^{-8}	3.42	44
GO:0051092	positive regulation of NF-kappaB transcription factor	BP	7.99×10^{-11}	3.80×10^{-8}	10.87	15
GO:0016567	protein ubiquitination	BP	8.43×10^{-11}	4.00×10^{-8}	5.62	25
GO:0070647	protein modification by small protein conjugation or	BP	8.64×10^{-11}	4.10×10^{-8}	4.83	29
GO:2001233	regulation of apoptotic signaling pathway	BP	2.59×10^{-10}	1.22×10^{-7}	6.83	20
GO:0051172	negative regulation of nitrogen compound metabolic	BP	2.98×10^{-10}	1.41×10^{-7}	3.06	48
GO:0006508	proteolysis	BP	3.07×10^{-10}	1.45×10^{-7}	3.71	37
GO:0042981	regulation of apoptotic process	BP	3.47×10^{-10}	1.63×10^{-7}	3.77	36
GO:2000058	regulation of ubiquitin-dependent protein catabolic	BP	3.76×10^{-10}	1.77×10^{-7}	12.15	13
GO:0044257	cellular protein catabolic process	BP	6.55×10^{-10}	3.07×10^{-7}	5.31	24
GO:1903362	regulation of cellular protein catabolic process	BP	6.72×10^{-10}	3.14×10^{-7}	8.56	16
GO:0010941	regulation of cell death	BP	7.08×10^{-10}	3.30×10^{-7}	3.53	38
GO:0051338	regulation of transferase activity	BP	9.26×10^{-10}	4.30×10^{-7}	4.36	29
GO:0006511	ubiquitin-dependent protein catabolic process	BP	9.25×10^{-10}	4.30×10^{-7}	6.35	20
GO:0071214	cellular response to abiotic stimulus	BP	9.33×10^{-10}	4.32×10^{-7}	7.73	17
GO:0032182	ubiquitin-like protein binding	MF	1.08×10^{-9}	4.97×10^{-7}	17.54	10
GO:0050790	regulation of catalytic activity	BP	1.09×10^{-9}	5.03×10^{-7}	3.23	42
GO:2001236	regulation of extrinsic apoptotic signaling pathway	BP	1.29×10^{-9}	5.95×10^{-7}	11.02	13
GO:2001238	positive regulation of extrinsic apoptotic signaling	BP	1.44×10^{-9}	6.59×10^{-7}	20.93	9
GO:0051603	proteolysis involved in cellular protein catabolic process	BP	1.71×10^{-9}	7.85×10^{-7}	5.53	22
GO:0014070	response to organic cyclic compound	BP	1.80×10^{-9}	8.21×10^{-7}	5.51	22
GO:0043085	positive regulation of catalytic activity	BP	2.04×10^{-9}	9.29×10^{-7}	3.88	32
GO:0010498	proteasomal protein catabolic process	BP	2.19×10^{-9}	9.97×10^{-7}	6.82	18
GO:0071260	cellular response to mechanical stimulus	BP	3.33×10^{-9}	1.51×10^{-6}	19.15	9
GO:0005102	signaling receptor binding	MF	3.48×10^{-9}	1.58×10^{-6}	3.46	36
GO:1901575	organic substance catabolic process	BP	3.87×10^{-9}	1.75×10^{-6}	3.38	37
GO:0071496	cellular response to external stimulus	BP	4.25×10^{-9}	1.92×10^{-6}	8.20	15
GO:0030154	cell differentiation	BP	5.03×10^{-9}	2.26×10^{-6}	3.17	40
GO:0018193	peptidyl-amino acid modification	BP	5.38×10^{-9}	2.41×10^{-6}	3.82	31
GO:0000151	ubiquitin ligase complex	CC	6.70×10^{-9}	3.00×10^{-6}	7.31	16
GO:0009894	regulation of catabolic process	BP	9.04×10^{-9}	4.04×10^{-6}	4.33	26
GO:0071345	cellular response to cytokine stimulus	BP	9.17×10^{-9}	4.09×10^{-6}	5.56	20
GO:0080135	regulation of cellular response to stress	BP	1.04×10^{-8}	4.63×10^{-6}	4.62	24
GO:0019901	protein kinase binding	MF	1.50×10^{-8}	6.67×10^{-6}	4.53	24
GO:1903322	positive regulation of protein modification by small protein conjugation or removal	BP	2.09×10^{-8}	9.25×10^{-6}	9.84	12
GO:0080134	regulation of response to stress	BP	2.28×10^{-8}	1.01×10^{-5}	3.51	32
GO:0098589	membrane region	CC	2.42×10^{-8}	1.07×10^{-5}	7.21	15
GO:0036462	TRAIL-activated apoptotic signaling pathway	BP	2.62×10^{-8}	1.15×10^{-5}	55.56	5
GO:0000165	MAPK cascade	BP	2.75×10^{-8}	1.21×10^{-5}	4.39	24
GO:0051347	positive regulation of transferase activity	BP	3.32×10^{-8}	1.46×10^{-5}	4.91	21
GO:0044248	cellular catabolic process	BP	3.93×10^{-8}	1.72×10^{-5}	3.04	38

GO:0044265	cellular macromolecule catabolic process	BP	4.04×10^{-8}	1.76×10^{-5}	4.02	26
GO:0018108	peptidyl-tyrosine phosphorylation	BP	4.13×10^{-8}	1.80×10^{-5}	6.43	16
GO:0097193	intrinsic apoptotic signaling pathway	BP	4.53×10^{-8}	1.97×10^{-5}	6.88	15
GO:0034599	cellular response to oxidative stress	BP	6.15×10^{-8}	2.66×10^{-5}	8.02	13
GO:1901698	response to nitrogen compound	BP	6.38×10^{-8}	2.76×10^{-5}	4.53	22
GO:0031329	regulation of cellular catabolic process	BP	6.50×10^{-8}	2.80×10^{-5}	4.35	23
GO:0071236	cellular response to antibiotic	BP	7.36×10^{-8}	3.16×10^{-5}	11.49	10
GO:0033993	response to lipid	BP	8.28×10^{-8}	3.55×10^{-5}	4.87	20
GO:0048468	cell development	BP	8.50×10^{-8}	3.64×10^{-5}	4.45	22
GO:1990234	transferase complex	CC	8.57×10^{-8}	3.66×10^{-5}	3.99	25
GO:0034976	response to endoplasmic reticulum stress	BP	1.01×10^{-7}	4.32×10^{-5}	7.69	13
GO:0045121	membrane raft	CC	1.26×10^{-7}	5.36×10^{-5}	6.90	14
GO:0051707	response to other organism	BP	1.34×10^{-7}	5.66×10^{-5}	4.34	22
GO:0010942	positive regulation of cell death	BP	1.62×10^{-7}	6.87×10^{-5}	4.47	21
GO:0043549	regulation of kinase activity	BP	1.65×10^{-7}	6.95×10^{-5}	4.13	23
GO:0070997	neuron death	BP	2.20×10^{-7}	9.27×10^{-5}	8.94	11
GO:2001235	positive regulation of apoptotic signaling pathway	BP	2.20×10^{-7}	9.27×10^{-5}	8.94	11
GO:0043066	negative regulation of apoptotic process	BP	2.36×10^{-7}	9.91×10^{-5}	4.20	22
GO:0061136	regulation of proteasomal protein catabolic process	BP	2.39×10^{-7}	1.00×10^{-4}	8.87	11
GO:0071797	LUBAC complex	CC	2.76×10^{-7}	1.15×10^{-4}	66.67	4
GO:0031398	positive regulation of protein ubiquitination	BP	2.79×10^{-7}	1.16×10^{-4}	10.00	10
GO:0044087	regulation of cellular component biogenesis	BP	2.86×10^{-7}	1.19×10^{-4}	3.75	25
GO:0045860	positive regulation of protein kinase activity	BP	3.00×10^{-7}	1.25×10^{-4}	5.23	17
GO:0032434	regulation of proteasomal ubiquitin-dependent protein catabolic process	BP	3.26×10^{-7}	1.35×10^{-4}	11.54	9
GO:0071396	cellular response to lipid	BP	4.06×10^{-7}	1.67×10^{-4}	5.12	17
GO:0051253	negative regulation of RNA metabolic process	BP	4.32×10^{-7}	1.78×10^{-4}	3.30	29
GO:0043408	regulation of MAPK cascade	BP	4.37×10^{-7}	1.80×10^{-4}	4.21	21
GO:0002684	positive regulation of immune system process	BP	4.42×10^{-7}	1.81×10^{-4}	3.90	23
GO:0051403	stress-activated MAPK cascade	BP	4.74×10^{-7}	1.94×10^{-4}	6.74	13
GO:0005126	cytokine receptor binding	MF	4.81×10^{-7}	1.96×10^{-4}	7.41	12
GO:0048545	response to steroid hormone	BP	4.81×10^{-7}	1.96×10^{-4}	7.41	12
GO:0006913	nucleocytoplasmic transport	BP	5.03×10^{-7}	2.05×10^{-4}	6.70	13
GO:0051248	negative regulation of protein metabolic process	BP	5.58×10^{-7}	2.26×10^{-4}	3.51	26
GO:0060548	negative regulation of cell death	BP	5.75×10^{-7}	2.33×10^{-4}	3.85	23
GO:0035690	cellular response to drug	BP	5.99×10^{-7}	2.42×10^{-4}	6.60	13
GO:0044388	small protein activating enzyme binding	MF	6.38×10^{-7}	2.57×10^{-4}	57.14	4
GO:0045862	positive regulation of proteolysis	BP	6.93×10^{-7}	2.79×10^{-4}	5.58	15
GO:2001242	regulation of intrinsic apoptotic signaling pathway	BP	7.06×10^{-7}	2.83×10^{-4}	7.97	11
GO:0043068	positive regulation of programmed cell death	BP	7.27×10^{-7}	2.91×10^{-4}	4.44	19
GO:1901222	regulation of NIK/NF-kappaB signaling	BP	7.59×10^{-7}	3.03×10^{-4}	10.47	9
GO:0045934	negative regulation of nucleobase-containing compound metabolic process	BP	7.76×10^{-7}	3.09×10^{-4}	3.07	31
GO:0008625	extrinsic apoptotic signaling pathway via death domain receptors	BP	8.05×10^{-7}	3.20×10^{-4}	12.50	8
GO:0034612	response to tumor necrosis factor	BP	9.72×10^{-7}	3.85×10^{-4}	6.94	12
GO:0031400	negative regulation of protein modification process	BP	1.06×10^{-6}	4.20×10^{-4}	4.33	19
GO:0044403	symbiont process	BP	1.11×10^{-6}	4.38×10^{-4}	4.76	17
GO:0009968	negative regulation of signal transduction	BP	1.22×10^{-6}	4.79×10^{-4}	3.28	27

GO:0071363	cellular response to growth factor stimulus	BP	1.24×10^{-6}	4.85×10^{-4}	4.49	18
GO:1905897	regulation of response to endoplasmic reticulum stress	BP	1.29×10^{-6}	5.05×10^{-4}	11.76	8
GO:0034614	cellular response to reactive oxygen species	BP	1.35×10^{-6}	5.26×10^{-4}	9.78	9
GO:0016477	cell migration	BP	1.96×10^{-6}	7.63×10^{-4}	3.13	28
GO:0044445	cytosolic part	CC	1.97×10^{-6}	7.66×10^{-4}	6.49	12
GO:0031647	regulation of protein stability	BP	2.39×10^{-6}	9.19×10^{-4}	5.41	14
GO:0045892	negative regulation of transcription. DNA-templated	BP	2.40×10^{-6}	9.20×10^{-4}	3.25	26
GO:0035821	modification of morphology or physiology of other	BP	2.38×10^{-6}	9.20×10^{-4}	7.05	11
GO:0051701	interaction with host	BP	2.39×10^{-6}	9.21×10^{-4}	7.94	10
GO:0051240	positive regulation of multicellular organismal process	BP	2.48×10^{-6}	9.52×10^{-4}	3.09	28
GO:0097435	supramolecular fiber organization	BP	2.80×10^{-6}	1.07×10^{-3}	4.45	17
GO:0033138	positive regulation of peptidyl-serine phosphorylation	BP	2.96×10^{-6}	1.13×10^{-3}	8.91	9
GO:1901214	regulation of neuron death	BP	2.96×10^{-6}	1.13×10^{-3}	8.91	9
GO:0006886	intracellular protein transport	BP	3.00×10^{-6}	1.14×10^{-3}	4.43	17
GO:0015031	protein transport	BP	3.56×10^{-6}	1.35×10^{-3}	3.26	25
GO:0045184	establishment of protein localization	BP	3.64×10^{-6}	1.38×10^{-3}	3.10	27
GO:0019221	cytokine-mediated signaling pathway	BP	4.00×10^{-6}	1.51×10^{-3}	6.06	12
GO:0010035	response to inorganic substance	BP	4.05×10^{-6}	1.52×10^{-3}	5.17	14
GO:0009895	negative regulation of catabolic process	BP	4.31×10^{-6}	1.61×10^{-3}	5.53	13
GO:0090322	regulation of superoxide metabolic process	BP	4.83×10^{-6}	1.81×10^{-3}	22.73	5
GO:0097342	ripoptosome	CC	5.79×10^{-6}	2.16×10^{-3}	36.36	4
GO:0032813	tumor necrosis factor receptor superfamily binding	MF	5.85×10^{-6}	2.17×10^{-3}	15.38	6
GO:0038083	peptidyl-tyrosine autophosphorylation	BP	6.12×10^{-6}	2.27×10^{-3}	21.74	5
GO:2000152	regulation of ubiquitin-specific protease activity	BP	6.41×10^{-6}	2.37×10^{-3}	75.00	3
GO:0051817	modification of morphology or physiology of other organism involved in symbiotic interaction	BP	6.98×10^{-6}	2.57×10^{-3}	8.04	9
GO:0042177	negative regulation of protein catabolic process	BP	7.51×10^{-6}	2.76×10^{-3}	7.96	9
GO:0032269	negative regulation of cellular protein metabolic process	BP	7.62×10^{-6}	2.80×10^{-3}	3.30	23
GO:1901701	cellular response to oxygen-containing compound	BP	7.72×10^{-6}	2.83×10^{-3}	3.64	20
GO:0051094	positive regulation of developmental process	BP	8.43×10^{-6}	3.08×10^{-3}	3.38	22
GO:1901700	response to oxygen-containing compound	BP	8.56×10^{-6}	3.12×10^{-3}	3.28	23
GO:0072593	reactive oxygen species metabolic process	BP	8.95×10^{-6}	3.25×10^{-3}	6.85	10
GO:0002682	regulation of immune system process	BP	9.23×10^{-6}	3.34×10^{-3}	3.09	25
GO:0072657	protein localization to membrane	BP	1.15×10^{-5}	4.17×10^{-3}	4.71	14
GO:0018105	peptidyl-serine phosphorylation	BP	1.17×10^{-5}	4.22×10^{-3}	5.45	12
GO:0008134	transcription factor binding	MF	1.21×10^{-5}	4.32×10^{-3}	3.66	19
GO:0043130	ubiquitin binding	MF	1.20×10^{-5}	4.32×10^{-3}	13.64	6
GO:2000059	negative regulation of ubiquitin-dependent protein catabolic process	BP	1.20×10^{-5}	4.32×10^{-3}	13.64	6
GO:0032183	SUMO binding	MF	1.23×10^{-5}	4.40×10^{-3}	30.77	4
GO:0009636	response to toxic substance	BP	1.28×10^{-5}	4.55×10^{-3}	6.58	10
GO:0033365	protein localization to organelle	BP	1.31×10^{-5}	4.64×10^{-3}	3.64	19
GO:0042493	response to drug	BP	1.31×10^{-5}	4.65×10^{-3}	4.16	16
GO:0019787	ubiquitin-like protein transferase activity	MF	1.36×10^{-5}	4.81×10^{-3}	4.37	15
GO:0017038	protein import	BP	1.40×10^{-5}	4.93×10^{-3}	7.38	9
GO:0031397	negative regulation of protein ubiquitination	BP	1.49×10^{-5}	5.24×10^{-3}	10.29	7
GO:0045732	positive regulation of protein catabolic process	BP	1.51×10^{-5}	5.30×10^{-3}	6.45	10
GO:0044003	modification by symbiont of host morphology or	BP	1.70×10^{-5}	5.94×10^{-3}	17.86	5
GO:0031264	death-inducing signaling complex	CC	1.71×10^{-5}	5.95×10^{-3}	28.57	4

GO:0051788	response to misfolded protein	BP	1.71×10^{-5}	5.95×10^{-3}	28.57	4
GO:0045595	regulation of cell differentiation	BP	1.72×10^{-5}	5.95×10^{-3}	3.14	23
GO:0002020	protease binding	MF	1.82×10^{-5}	6.28×10^{-3}	7.14	9
GO:0010243	response to organonitrogen compound	BP	1.98×10^{-5}	6.82×10^{-3}	4.02	16
GO:0016234	inclusion body	CC	2.04×10^{-5}	7.01×10^{-3}	8.16	8
GO:0042826	histone deacetylase binding	MF	2.20×10^{-5}	7.53×10^{-3}	8.08	8
GO:0051702	interaction with symbiont	BP	2.20×10^{-5}	7.53×10^{-3}	8.08	8
GO:1903364	positive regulation of cellular protein catabolic process	BP	2.20×10^{-5}	7.53×10^{-3}	8.08	8
GO:0043254	regulation of protein complex assembly	BP	2.23×10^{-5}	7.62×10^{-3}	4.44	14
GO:0005164	tumor necrosis factor receptor binding	MF	2.31×10^{-5}	7.88×10^{-3}	26.67	4
GO:0005615	extracellular space	CC	2.48×10^{-5}	8.45×10^{-3}	3.15	22
GO:0033209	tumor necrosis factor-mediated signaling pathway	BP	2.61×10^{-5}	8.85×10^{-3}	9.46	7
GO:0001819	positive regulation of cytokine production	BP	2.84×10^{-5}	9.60×10^{-3}	4.35	14
GO:0007250	activation of NF-kappaB-inducing kinase activity	BP	3.05×10^{-5}	1.03×10^{-2}	25.00	4
GO:1990381	ubiquitin-specific protease binding	MF	3.05×10^{-5}	1.03×10^{-2}	25.00	4
GO:0005114	type II transforming growth factor beta receptor binding	MF	3.15×10^{-5}	1.06×10^{-2}	50.00	3
GO:0043256	laminin complex	CC	3.15×10^{-5}	1.06×10^{-2}	50.00	3
GO:0072717	cellular response to actinomycin D	BP	3.15×10^{-5}	1.06×10^{-2}	50.00	3
GO:0001817	regulation of cytokine production	BP	3.16×10^{-5}	1.06×10^{-2}	3.70	17
GO:0006952	defense response	BP	3.43×10^{-5}	1.15×10^{-2}	3.18	21
GO:1902532	negative regulation of intracellular signal transduction	BP	3.48×10^{-5}	1.16×10^{-2}	3.84	16
GO:0006974	cellular response to DNA damage stimulus	BP	3.78×10^{-5}	1.25×10^{-2}	3.26	20
GO:0071248	cellular response to metal ion	BP	3.86×10^{-5}	1.28×10^{-2}	7.48	8
GO:0016032	viral process	BP	3.90×10^{-5}	1.29×10^{-2}	4.50	13
GO:1900407	regulation of cellular response to oxidative stress	BP	4.00×10^{-5}	1.32×10^{-2}	11.11	6
GO:0009725	response to hormone	BP	4.24×10^{-5}	1.39×10^{-2}	4.19	14
GO:0045597	positive regulation of cell differentiation	BP	4.25×10^{-5}	1.39×10^{-2}	3.77	16
GO:0000122	negative regulation of transcription by RNA polymerase	BP	4.44×10^{-5}	1.45×10^{-2}	3.45	18
GO:2001243	negative regulation of intrinsic apoptotic signaling	BP	4.70×10^{-5}	1.53×10^{-2}	8.64	7
GO:0046907	intracellular transport	BP	4.78×10^{-5}	1.55×10^{-2}	3.02	22
GO:0010038	response to metal ion	BP	4.98×10^{-5}	1.61×10^{-2}	5.62	10
GO:0070513	death domain binding	MF	5.04×10^{-5}	1.62×10^{-2}	22.22	4
GO:0043491	protein kinase B signaling	BP	5.23×10^{-5}	1.68×10^{-2}	6.25	9
GO:0004062	aryl sulfotransferase activity	MF	5.46×10^{-5}	1.75×10^{-2}	42.86	3
GO:0051170	import into nucleus	BP	5.95×10^{-5}	1.90×10^{-2}	8.33	7
GO:0045321	leukocyte activation	BP	6.02×10^{-5}	1.91×10^{-2}	4.06	14
GO:0070266	necroptotic process	BP	6.05×10^{-5}	1.92×10^{-2}	13.89	5
GO:1901699	cellular response to nitrogen compound	BP	6.21×10^{-5}	1.96×10^{-2}	4.05	14
GO:0044390	ubiquitin-like protein conjugating enzyme binding	MF	6.32×10^{-5}	1.99×10^{-2}	21.05	4
GO:0000902	cell morphogenesis	BP	6.77×10^{-5}	2.13×10^{-2}	4.26	13
GO:0046330	positive regulation of JNK cascade	BP	6.87×10^{-5}	2.15×10^{-2}	6.90	8
GO:0004672	protein kinase activity	MF	7.75×10^{-5}	2.42×10^{-2}	3.59	16
GO:0043410	positive regulation of MAPK cascade	BP	7.93×10^{-5}	2.47×10^{-2}	3.95	14
GO:0052547	regulation of peptidase activity	BP	8.13×10^{-5}	2.52×10^{-2}	4.48	12
GO:0001085	RNA polymerase II transcription factor binding	MF	8.24×10^{-5}	2.55×10^{-2}	6.72	8
GO:0072594	establishment of protein localization to organelle	BP	8.36×10^{-5}	2.57×10^{-2}	4.82	11
GO:1903351	cellular response to dopamine	BP	8.66×10^{-5}	2.66×10^{-2}	37.50	3
GO:1903800	positive regulation of production of miRNAs involved in gene silencing by miRNA	BP	8.66×10^{-5}	2.66×10^{-2}	37.50	3

GO:0036473	cell death in response to oxidative stress	BP	8.80×10^{-5}	2.69×10^{-2}	9.68	6
GO:0051341	regulation of oxidoreductase activity	BP	9.63×10^{-5}	2.94×10^{-2}	9.52	6
GO:0010952	positive regulation of peptidase activity	BP	9.73×10^{-5}	2.96×10^{-2}	5.77	9
GO:0009896	positive regulation of catabolic process	BP	1.06×10^{-4}	3.22×10^{-2}	4.08	13
GO:0050730	regulation of peptidyl-tyrosine phosphorylation	BP	1.07×10^{-4}	3.23×10^{-2}	5.13	10
GO:0035966	response to topologically incorrect protein	BP	1.15×10^{-4}	3.46×10^{-2}	9.23	6
GO:0045672	positive regulation of osteoclast differentiation	BP	1.29×10^{-4}	3.86×10^{-2}	33.33	3
GO:0051900	regulation of mitochondrial depolarization	BP	1.29×10^{-4}	3.86×10^{-2}	33.33	3
GO:0051099	positive regulation of binding	BP	1.36×10^{-4}	4.07×10^{-2}	5.52	9
GO:2001234	negative regulation of apoptotic signaling pathway	BP	1.36×10^{-4}	4.07×10^{-2}	5.52	9
GO:0030225	macrophage differentiation	BP	1.39×10^{-4}	4.15×10^{-2}	17.39	4
GO:0002718	regulation of cytokine production involved in immune response	BP	1.45×10^{-4}	4.29×10^{-2}	11.63	5
GO:0002824	positive regulation of adaptive immune response based on somatic recombination of immune receptors built from immunoglobulin superfamily domains	BP	1.45×10^{-4}	4.29×10^{-2}	11.63	5
GO:0051881	regulation of mitochondrial membrane potential	BP	1.45×10^{-4}	4.29×10^{-2}	11.63	5
GO:1903573	negative regulation of response to endoplasmic reticulum stress	BP	1.45×10^{-4}	4.29×10^{-2}	11.63	5
GO:0031330	negative regulation of cellular catabolic process	BP	1.49×10^{-4}	4.40×10^{-2}	4.93	10
GO:0046777	protein autophosphorylation	BP	1.55×10^{-4}	4.57×10^{-2}	4.90	10
GO:0050776	regulation of immune response	BP	1.69×10^{-4}	4.96×10^{-2}	3.50	15

MF (molecular function), BP (biological process), CC (cellular component)

Supplementary Table S13. GO analysis of the extended interactome of integrated blood markers linked to the long-term anti-TNF response in CD patients.

GO ID	GO term	ontology source	p-value	adjusted p-value	% assoc. genes	nr. assoc. genes
GO:0005874	microtubule	CC	1.73×10^{-9}	1.90×10^{-8}	5.29	10
GO:0070507	regulation of microtubule cytoskeleton organization	BP	2.09×10^{-7}	2.09×10^{-6}	4.71	8
GO:0051010	microtubule plus-end binding	MF	1.07×10^{-5}	9.63×10^{-5}	25.00	3
GO:0008017	microtubule binding	MF	3.69×10^{-5}	2.96×10^{-4}	4.95	5
GO:0031110	regulation of microtubule polymerization or depolymerization	BP	4.86×10^{-5}	3.40×10^{-4}	7.41	4
GO:0004843	thiol-dependent ubiquitin-specific protease activity	MF	2.48×10^{-4}	1.49×10^{-3}	4.88	4
GO:0019887	protein kinase regulator activity	MF	8.12×10^{-4}	2.44×10^{-3}	3.57	4
GO:0072698	protein localization to microtubule cytoskeleton	BP	5.53×10^{-4}	2.76×10^{-3}	6.98	3
GO:0016579	protein deubiquitination	BP	7.85×10^{-4}	3.14×10^{-3}	3.60	4
GO:1902554	serine/threonine protein kinase complex	CC	3.83×10^{-3}	3.83×10^{-3}	3.57	3
GO:0042102	positive regulation of T cell proliferation	BP	2.38×10^{-3}	4.76×10^{-3}	4.23	3

MF (molecular function), BP (biological process), CC (cellular component)
